# Quercetin 7-rhamnoside protects against alpha-naphthylisothiocyanate (ANIT)-induced in cholestatic hepatitis rats by improving biliary excretion and inhibiting inflammatory responses

**DOI:** 10.3389/fphar.2022.1116257

**Published:** 2023-01-09

**Authors:** Hong-Liu Jin, Xiao-Jia Liu, Xiao-Ying Feng, Wen-Ting Zhu, Sen-Ling Feng, Li-Ping Cao, Zhong-Wen Yuan

**Affiliations:** ^1^ Department of Pharmacy, The Third Affiliated Hospital of Guangzhou Medical University, Guangzhou, China; ^2^ School of Pharmaceutical Sciences, Guangzhou Medical University, Guangzhou, China; ^3^ Guangdong Provincial Key Laboratory of Major Obstetric Diseases, Guangzhou Medical University, Guangzhou, China; ^4^ School of Pharmaceutical Sciences, Guangzhou University of Chinese Medicine, Guangzhou, China; ^5^ Department of Pharmacy, Shenzhen Bao’an Traditional Chinese Medicine Hospital Group, Guangzhou University of Chinese Medicine, Shenzhen, China

**Keywords:** quercetin 7-rhamnoside, cholestatic hepatitis, inflammation, bile acid, traditional Chinese medicine

## Abstract

**Objective:** To explore the pharmacological effects and molecular mechanism of quercetin 7-rhamnoside (Q7R) in the treatment of cholestatic hepatitis induced by alpha-naphthylisothiocyanate (ANIT).

**Methods:** ANIT-induced cholestatic hepatitis rat model was used to investigate the hepatoprotective effects of three different doses of Q7R (1.25 mg/kg; 2.5 mg/kg; 5 mg/kg). Serum biochemical indices were detected using commercial kits. H&E and masson staining were used to observe hepatic tissue damage and collagen deposition in hepatocytes. The metabolism of bile acid-related substances was detected *via* HPLC-MS/MS by 5-(diisopropylamino) amylamine (DIAAA) derivative method. Hepatocyte injury, cholestasis, and inflammation were detected at the mRNA and protein levels using reverse transcription-polymerase chain reaction (RT-PCR) and western blotting, respectively.

**Results:** Q7R can decrease the level of CYP7A1, and increase FXR, CYP27A1 so then improving abnormal bile acid secretion. Furthermore, Q7R can also ameliorating inflammation by reduce TNF-α, IL-1β, PTGS1, PTGS2, NCOA2, NF-κB level. Therefore, Q7R had an effective therapeutic effect on ANIT-induced cholestatic hepatitis, improving abnormal bile acid secretion, and inhibiting inflammatory responses.

**Conclusion:** The results demonstrated that Q7R treat cholestatic hepatitis by regulating bile acid secretion and alleviating inflammation.

## 1 Introduction

The liver is the most important organ for the excretion of cholesterol from the body. Cholestasis is a common clinical disorder of intrahepatic cholesterol metabolism and bile acid metabolism that causes serious damage to the liver. A large amount of cholesterol is first converted into bile acids in the liver before being discharged from the body (Wang et al., 2018). Bile acid has important physiological functions that can reduce the risk for cardiovascular diseases caused by high cholesterol, fatty liver, and cirrhosis. Therefore, bile acid synthesis is vital for maintaining normal cholesterol levels (Wagner et al., 2009). Alpha-naphthylisothiocyanate (ANIT) is a hepatotoxic agent that causes cholestatic hepatitis. It mainly damages intrahepatic bile duct epithelial cells and causes capillary bile duct hyperplasia and inflammation around the interlobular bile duct, resulting in bile duct obstruction and cholestasis. Moreover, it is accompanied by spotty necrosis of the liver and parenchymal cell damage, resulting in cholestatic jaundice, hyperbilirubinemia, and decreased bile secretion. Therefore, it is widely used to induce cholestatic hepatitis in related researc (Roth and Dahm, 1997; Chen et al., 2021).

Traditional Chinese medicine (TCM) are used for treatment of cholestatic hepatitis with a long history. There have been many pharmacology and molecular mechanism researches on Chinese herbal medicine treat cholestatic hepatitis. Wang et al. (Wang et al., 2022) explored the therapeutic effect of Da-Huang-Xiao-Shi Decoction on cholestasis; Wu et al. (Wu et al., 2022) explored the therapeutic effect of combination of resveratrol and luteolin on cholestasis by regulating bile acid homeostasis and inhibiting oxidative stress to improve disease occurrence. *Hypericum japonicum* is a dried whole plant of *Hypericum japonicum* Thunb., and has been widely applied to cure infectious hepatitis, acute and chronic hepatitis in traditional medicine. In addition, flavonoids, phloroglucinols, phenolic acids and xanthones and trimethylated acylphloroglucinol meroterpenoids isolated from *Hypericum japonicum* Thunb. have already been proved the hepatoprotective, anti-tumor, antibacterial, antiviral, and antioxidant activities (Liu et al., 2014; Peron et al., 2019; Deng et al., 2022).

Our previous research also proved that *Hypericum japonicum* has the effect of improving cholestatic hepatitis (Feng et al., 2021). The quercetin 7-rhamnoside (Q7R) is main flavonoid component of *Hypericum japonicum*, which has strong hepatoprotective, antioxidant, and antiviral activity (Choi et al., 2009; Song et al., 2011). Awaad et al. (Awaad et al., 2006) found that Q7R has therapeutic effect on CCl_4_ induced liver fibrosis, and Li et al. (Liang et al., 2013) also explored the effect of Q7R on the apoptosis of L-O2 cells induced by glycodeoxycholic acid, and confirmed that Q7R can inhibit the excessive production of ROS and GSH depletion, decrease the production of malonaldehyde and increased catalase activities, and then reducing the apoptosis of normal hepatocytes (Huang et al., 2018).

In this research, we used a rat model of ANIT-induced cholestatic hepatitis to investigate the effects of Q7R on bile acid metabolism and inflammation. The therapeutic effect of Q7R on cholestatic hepatitis was verified using pharmacological experiments of pathological evaluation and molecular mechanism.

## 2 Materials and methods

### 2.1 Materials and chemicals

Quercetin 7-rhamnoside was purchased from Biopurify (Chengdu Biopurify Phytochemicals Ltd.). Kits for detecting albumin (ALB), aspartate aminotransferase (AST), and alanine aminotransferase (ALT) were purchased from Nanjing Jiancheng (Nanjing Jiancheng Bioengineering Institute). Ursodeoxycholic acid (>98%, J0324A), Chenodeoxycholic acid (>98%, D1217A), Cholic acid (>98%, M0412AS), Hyodeoxycholic acid (>98%, D1017AS), Deoxycholic acid (>98%, J0602AS) were purchased from meilunbio (Dalian Meilun Biotech Co., Ltd.). d4-glycochenodeoxycholate (>99.5%, ZZS19022701) was purchased from ZZBIO (Shanghai ZZBIO Co., Ltd.). 5-(Diisopropylamino) amylamine (DIAAA, B02669513), 1-[Bis (dimethylamino) methylene]-1H-1,2,3-triazolo [4,5-b] pyridinium 3-oxid hexafluorophosphate (HATU,≥98.0%,102409029), 1-Hydroxybenzotriazole hydrate (HOBt, ≥97.0%, BCCF3867), Triethylamine (TEA, ≥99.5%) were purchased from Sigma (Sigma-Aldrich (Shanghai) Trading Co. Ltd.). Primary anti-FXR antibodies were purchased from Abcam (Abcam Plc.). anti-CYP7A1 antibodies were purchased from Boster (Boster Biological Technology Co., Ltd.), and anti-CYP27A1 antibodies were purchased from Affinity Biosciences (Affinity Biosciences Co., Ltd.). Secondary antibodies such as goat anti-mouse IgG (H + L) and goat anti-rabbit IgG (H + L) were purchased from CWBIO (Beijing Kangwei Century Biotechnology Co., Ltd.).

### 2.2 Animals

Male Sprague-Dawley rats (SD) rats (200–220 g) were purchased from Guangzhou Ruige Biological Technology Co., Ltd. (medical experimental animal number: SCXK [YUE] 2021-0059; animal certification number: 44827200001399). All animals were acclimatized and kept in a well-ventilated environment at 22–25°C and 50%–60% relative humidity under a 12/12 h light/dark cycle for 1 week. Rats were allowed free access to food and water and were fasted for 1 day prior to the experiments. All animal care and experimental protocols were performed according to the guidelines for the Institutional Animal Care and Use of Laboratory Animals at Guangzhou Medical University (Ethical approval: 2018-051).

### 2.3 Experimental design

**FIGURE 1:**
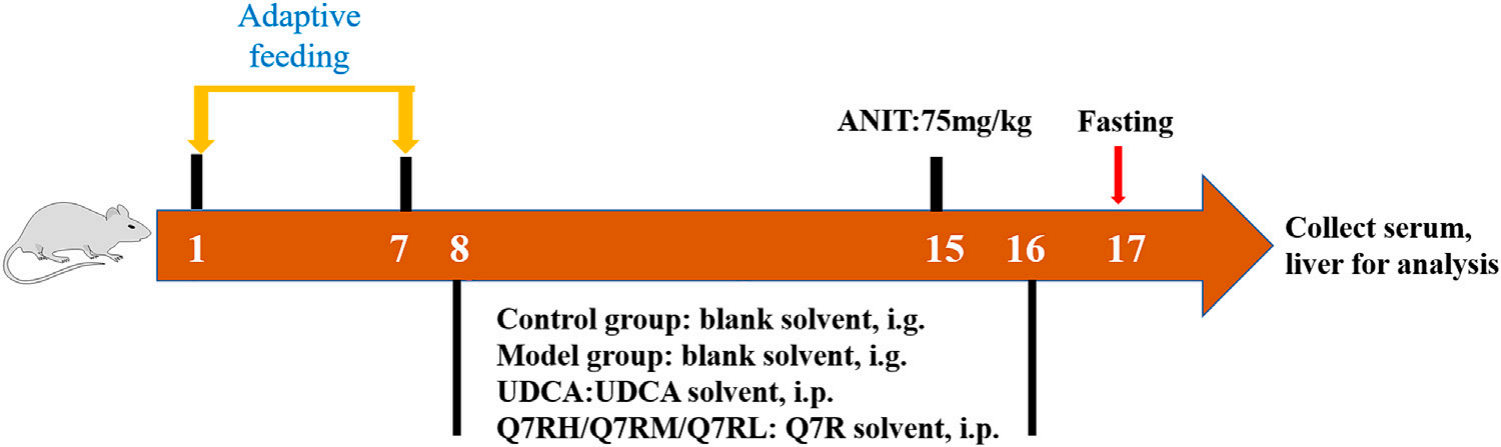
Diagrammatic sketch of the experimental design of the effect of Q7R on rat cholestatic hepatitis.

To evaluate the therapeutic effects of Q7R, thirty-six rats were randomly divided into six groups (*n* = 6 rats each group): 1) control group and 2) model group: blank solvent, intragastrical administration (i.g.); (3) positive control group: ursodeoxycholic acid (UDCA) 50 mg/kg, i.g.; 4) high-dose group (Q7RH): 5 mg/kg Q7R, i.p.; 5) medium-dose group (Q7RM): 2.5 mg/kg Q7R, i.p.; and 6) low-dose group (Q7RL) 1.25 mg/kg, i.p. ANIT was administered 1 week later in all groups (Figure 1).

### 2.4 Sample collection and liver index calculation

Animals were fasted for 24 h after the last administration. Before the rats in each group were anesthetized with pentobarbital sodium (60 mg/kg) and sacrificed, their body weights were recorded. Subsequently, blood was collected from the abdominal aorta for biochemical assessment, and livers were isolated and accurately weighed after the remaining blood on their surface was washed with PBS and wiped clean with filter paper. The formula for calculating the relative liver index was as follows: Liver index = Liver weight/Rat body weight × 100%.

### 2.5 Histopathology and biochemical analysis

The isolated rat livers were fixed with 4% paraformaldehyde, embedded in paraffin, and sliced. Slices were stained with hematoxylin and eosin (H&E), and masson-stained. Histological changes were evaluated by randomly selecting visual fields using an upright microscope (Nikon, Japan). The levels of alanine transaminase (ALT), aspartate transaminase (AST), gamma-glutamyl transferase (γ-GGT), total bilirubin (TBIL) and direct bilirubin (DBIL) were measured using a Chemray 240 Automatic Biochemical Analyzer (Rayto Life and Analytical Sciences Co., Ltd., Shenzhen, China). Albumin (ALB) was evaluated using commercial kits, according to the manufacturer’s instructions.

### 2.6 Reverse transcription-polymerase chain reaction (RT-PCR)

Total RNA was extracted from liver tissue using an RNA extraction solution (Wuhan Servicebio Biotechnology Co., Ltd. Wuhan, China) according to the manufacturer’s instructions. The primer sequences used for PCR are listed in Table 1. Briefly, total RNA (10 µL) was reverse transcribed into cDNA. Each PCR reaction (20 µL) for prostaglandin G/H synthase 1 (PTGS1), prostaglandin G/H synthase 2 (PTGS2), nuclear receptor coactivator 2 (NCOA2), interleukin-1β (IL-1β), nuclear factor kappa-B (NF-κB), farnesoid X receptor (FXR), and tumor necrosis factor-α (TNF-α) included 2 µL cDNA, 1.5 µL of each primer (2.5 µM), 7.5 µL of 2× qPCR Mix, and 4 µL nuclease-free water. RT-PCR was performed as follows: pre-denaturation at 95°C for 30 s, followed by 40 cycles of denaturation at 95°C for 15 s, and annealing at 60°C for 30 s. The melting curve was obtained from 65°C to 95°C, and fluorescence signals were acquired for every .5°C increase in temperature. The expression of *PTGS1*, *PTGS2*, *NCOA2*, *IL-1β*, *NF-κB*, *FXR*, and *TNF-*α mRNAs relative to *GAPDH* mRNA expression was detected using the 2^−ΔΔCT^ method.

**TABLE 1:** Primers for real-time reverse transcription-polymerase chain reaction (RT-PCR).

Gene	Primer sequences (5′-3′)	Fragment length (bp)
PTGS1	GGT​CTG​ATG​CTC​TTC​TCC​ACG	135
	GAT​GGT​TTC​CCC​TAT​AAG​GAT​GA	
FXR	CCA​TTT​ACA​AGC​CAC​GGA​CG	180
	CCC​AGG​TTG​GAA​TAA​TAG​GAC​G	
NCOA2	AAC​CAG​CCA​AAC​CAA​CTG​AGA​C	270
	GGT​CAT​ATT​CAA​CCC​TTG​TCC​TCT	
PTGS2	GTG​TTG​ACG​TCC​AGA​TCA​CAT​TTG	132
	GCA​GTC​ATC​AGC​CAC​AGG​AG	
TNF-α	CCA​GGT​TCT​CTT​CAA​GGG​ACA​A	80
	GGT​ATG​AAA​TGG​CAA​ATC​GGC​T	
IL-1β	GAA​CAA​CAA​AAA​TGC​CTC​GTG​C	264
	GAC​AAA​CCG​CTT​TTC​CAT​CTT​CT	
NF-κB	GGC​CCA​TAC​CTT​CAA​ATA​CTA​GAG​C	130
	CCT​GCG​GGT​AAG​ATT​TCT​TGT​TC	
GAPDH	CTG​GAG​AAA​CCT​GCC​AAG​TAT​G	138
	GGT​GGA​AGA​ATG​GGA​GTT​GCT	

### 2.7 Western blotting

The proteins in liver tissue of rats in each group were extracted and quantified using the BCA method. The protein samples were then subjected to electrophoresis, membrane transfer, blocking, and incubation with anti-FXR (1:1000), anti-CYP7A1 (1:1000), anti-CYP27A1 (1:1000) antibodies. The membranes were washed three times with TBST and incubated with the corresponding horseradish peroxidase-labeled secondary antibodies (1:3000) for 1 h. After washing three times with TBST, the proteins were detected using an ECL chemiluminescence reagent. After exposure scanning (Bio-Rad Laboratories, Hercules, CA, United States), ImageJ software was used for analysis.

### 2.8 Detection of bile acid metabolites (BAs) in Serum by high-performance liquid chromatography-mass spectrometry (HPLC-MS/MS)

Serum (100 μL) was added to 400 μL of cold methanol (100 ng/mL of D4-GCA), vortexing for 1 min, the precipitated protein was centrifuged (5804R, Eppendorf, Hamburg, Germany) at 21380 g at 4°C for 15 min, and 400 μL supernatant was collected and repeated three times. The 1200 μL supernatant was combined and vacuum dried. 5 μL HOBt, 5 μL DIAAA-TEA, 5 μL HATU were successively added to the dried product, vortexed for 1 min, then incubated at room temperature for 2 min, add 35 μL acetonitrile, vortexed, centrifuged at 21,380 g for 15 min at 4°C, and collected 40 μL of the supernatant for analysis (Bian et al., 2018).

Serum extracts were analyzed on a Sciex 4000 Triple Quadrupole HPLC-MS/MS system (AB Sciex, Massachusetts, United States) using a Symmetry C18 analytical column (3.5 μm, 2.1 × 50 mm; Waters Co., Milford, MA, United States). The mobile phase containing 1% formic acid aqueous solution (solvent A) and methanol solution (solvent B) was provided according to the following procedure: 0–8 min (5%–40% B); 8–15 min (40%–60% B); 15–18 min (60%–80% B); 18–20 min (80%–98% B); 20–25 min (98%–60% B); 25–30 min (60%–40% B). The injection volume and flow rate were 10 μL and 25 mL/min, respectively. The multiple reaction monitoring parameters were listed in Table 2.

**TABLE 2:** Mass spectrometric characteristics of CA-DIAAA, CDCA-DIAAA, DCA-DIAAA, UDCA-DIAAA, HDCA-DIAAA, and IS-DIAAA (D4-GCA-DIAAA).

Compound	Q1 Mass (Da)	Q3 Mass (Da)	DP (volts)	EP(Volts)	CE (volts)	CXP (volts)
CA-DIAAA	577.50	577.50	138.00	10.00	33.04	5.38
CDCA-DIAAA	561.30	561.30	107.76	10.62	32.52	5.43
DCA-DIAAA	561.40	561.40	105.50	9.81	32.52	5.80
UDCA-DIAAA	561.40	561.30	103.20	10.28	32.52	5.07
HDCA- DIAAA	561.40	561.40	107.43	11.20	32.52	5.56
(D4-GCA-DIAAA) IS	622.50	622.50	121.76	11.35	34.5	5.89

Note: CA-DIAAA, derivatization of cholic acid; CDCA-DIAAA, derivatization of chenodeoxycholic acid; DCA-DIAAA, derivatization of deoxycholic acid; UDCA-DIAAA, derivatization of ursodeoxycholic; HDCA-DIAAA, derivatization of hyodeoxycholic acid; IS, internal standard; D4-GCA-DIAAA, derivatization of D4-glycocholic acid.

The following MS parameters were used: curtain gas = 35 psi; ion spray voltage = +4500 V; temperature = 450°C; ion source gas 1 = 45 psi; ion source gas 2 = 45 psi; interface heater = on; collision gas = medium. The multiple reaction monitoring parameters are listed in Table 2. All of the HPLC-MS/MS data were obtained using Analyst software (v1.6.2).

### 2.9 Statistical analysis

All experimental data are expressed as the mean ± standard error (SEM). Statistical analyses were performed using GraphPad Prism 9.0 software. Mean comparisons between two data sets were performed using the *t*-test, and mean comparisons between three or more treatments were performed using one-way analysis of variance. *p* < .05 was considered statistically significant.

## 3 Results

### 3.1 Protective effect of Q7R on cholestatic hepatitis in rats

The liver index of the model group was higher than that of the control group. The liver indices of the positive control, Q7RM, and Q7R H groups were all significantly decreased (*p < .05*) compared to that of the model group (Supplementary Figure S1).

**FIGURE 2:**
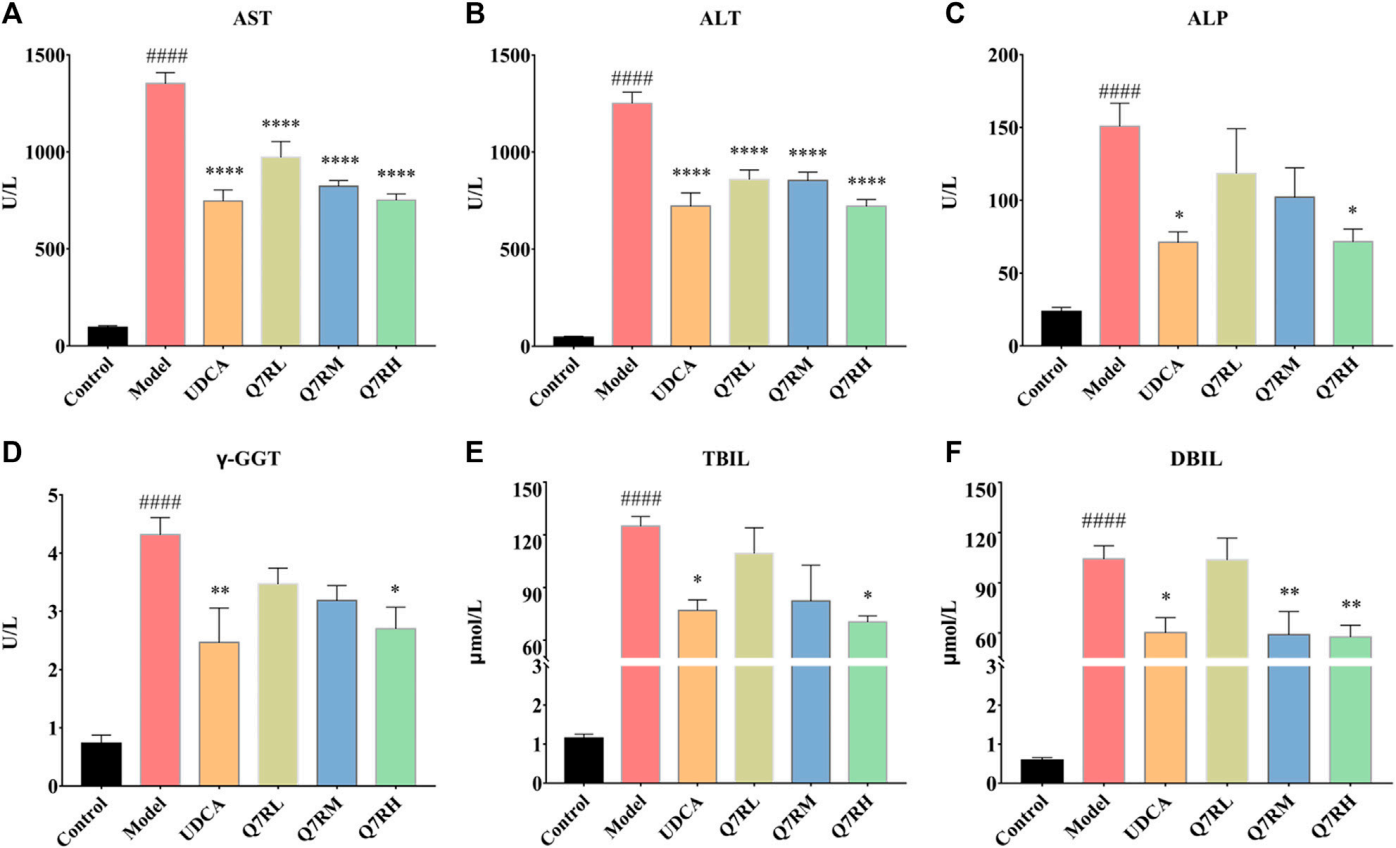
Effect of Q7R treatment on the levels of serum biochemical indices in model rats (*n* = 6). **(A)** Serum AST; **(B)** Serum ALT; **(C)** Serum ALP; **(D)** Serum γ-GGT; **(E)** Serum TBIL; **(F)** Serum DBIL; Values are presented as mean ± SEM for three biological replicates per group. *p < .05, **p < .01, ****p < .001 vs. the model group, ^####^ *p* < .001 vs. the control group.

Biochemical analys was conducted to evaluate the therapeutic effects of Q7R. Results showed that the levels of AST, ALT, ALP, γ-GGT, TBIL, and DBIL in the model group were significantly increased (*p* < .05) (Figure 2). In contrast, the levels of these parameters were decreased significantly (*p* < .05) in the positive control group and the different Q7R-treatment groups.

### 3.2 Q7R alleviates cholestatic hepatitis

**FIGURE 3:**
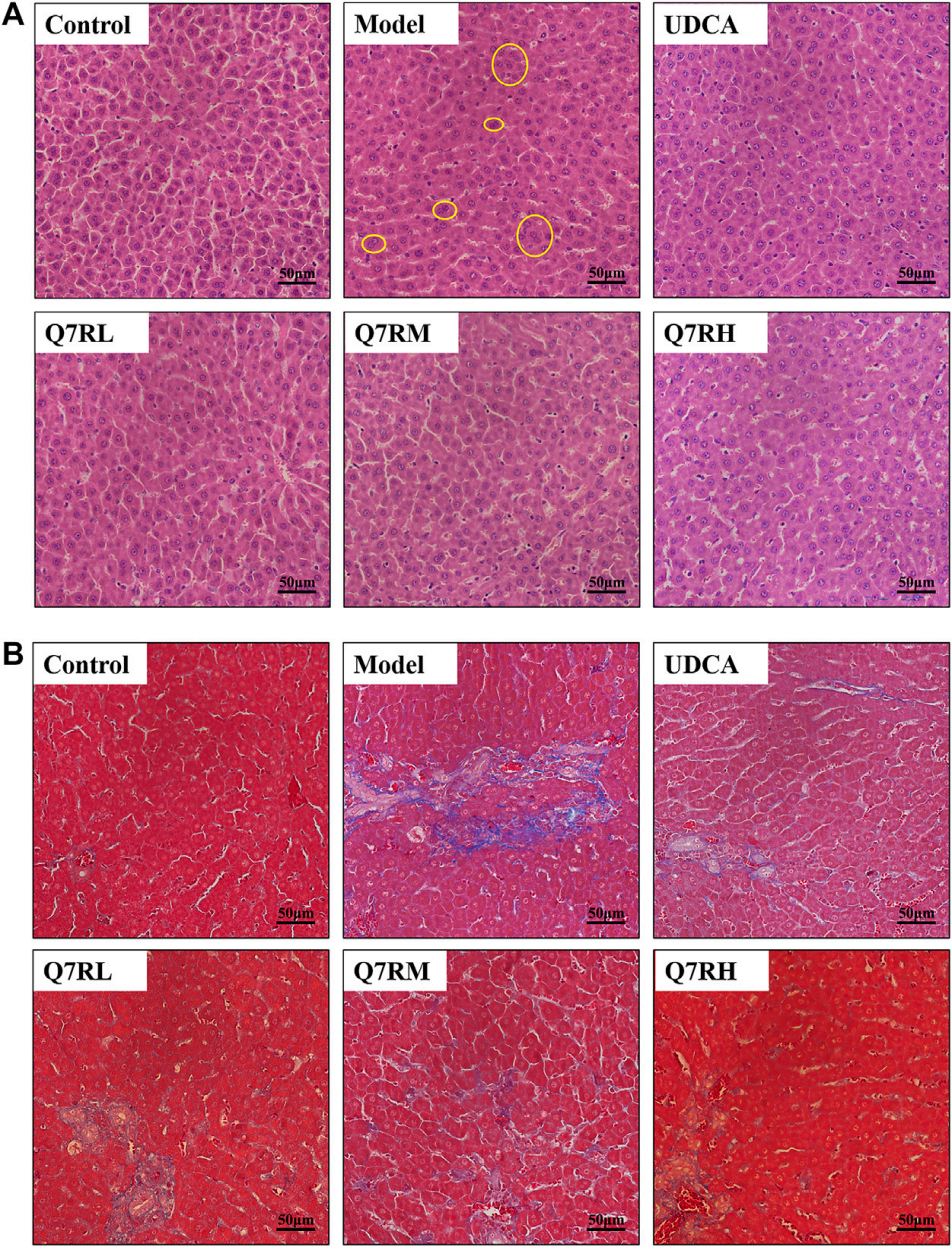
Histological analysis. **(A)** Hematoxylin and eosin (H&E)-stained sections of the liver. Original magnification × 40. **(B)** Masson-stained sections of the liver. Original magnification × 40. The part circled by yellow circle is inflammatory cell infiltration.

The results of H&E staining (Figure 3A) showed that the structure of the hepatocytes in the blank control group is normal, and the hepatic lobules was complete, the cells sizes were uniform, their nucleus were clearly visible, without cell necrosis or the infiltration of inflammatory cell. In contrast, the sizes of the hepatocytes in the model group were different, and their cytoplasms were loose. The liver cells also showed focal necrosis, eosinophilic changes, and punctate necrosis. Inflammatory factor infiltration was also observed in the liver tissue. The positive control and high-dose groups were significantly lighter than the model group, and the low-dose group also improved. This finding showed that the drug effectively improved liver tissue cell damage.

Masson staining (Figure 3B) showed that the cell structure of liver tissue sections in the blank control group was complete, and no obvious blue collagen fiber deposition was observed. However, in the liver tissue sections of the model group, abnormal structural hepatic lobules and hepatocyte degeneration and necrosis were observed. The appearance of a large amount of blue collagen fiber deposition in the liver indicates serious fibrosis. The Area of collage fibers in liver according to masson stain was shown in Supplementary Figure S2.

Q7R treatment significantly attenuated the degree of hepatic putrescence, fibrosis, and inflammatory cell infiltration. These results revealed that Q7R protected against ANIT-induced liver fibrosis in cholestatic hepatitis rats.

### 3.3 Effect of Q7R on inflammation and bile acid secretion

**FIGURE 4:**
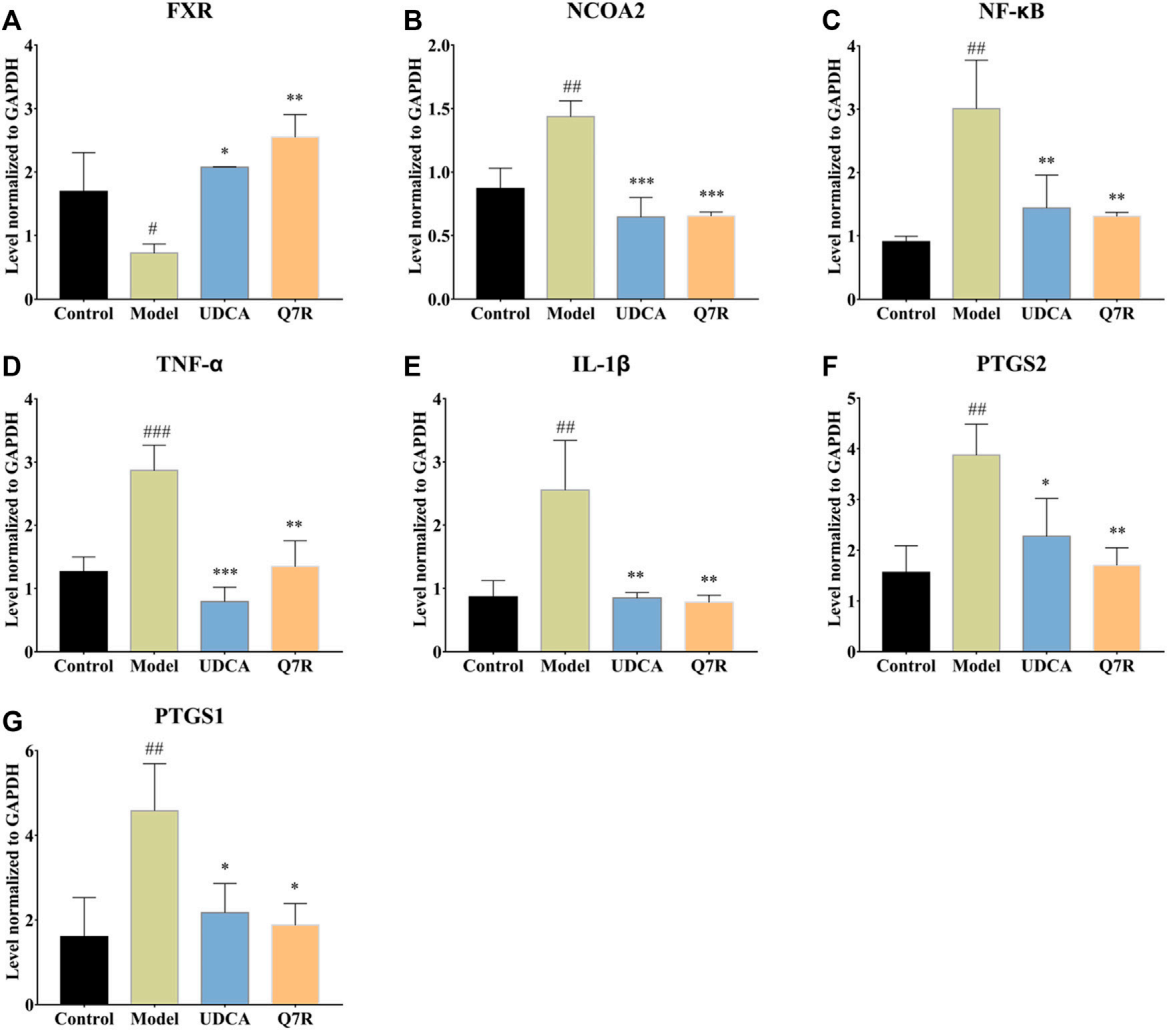
RT-PCR analysis for FXR, NCOA2, NF-κB, TNF-α, IL-1β, PTGS2, PTGS1 mRNA levels in the liver of model rats (*n* = 3). **(A)** FXR; **(B)** NCOA2; **(C)** NF-κB; **(D)** TNF-α; **(E)** IL-1β; **(F)** PTGS2; **(G)** PTGS1; Values are presented as mean ± SEM for three biological replicates per group. *p < .05, **p < .01, ***p < .005 vs. the model group, ^#^ *p* < .05, ^##^ *p* < .01, ^###^ *p* < .005 vs. the control group.

To evaluate the effect of Q7R treatment on bile acid secretion and inflammation, mRNA levels of *TNF-*α, *IL-1β*, *PTGS1*, *PTGS2*, *NCOA2*, *FXR*, and *NF-κB* were measured. The results showed that the mRNA expression of *TNF-*α, *IL-1β*, *PTGS1*, *PTGS2*, *NCOA2*, and *NF-κB* were upregulated in the model group but were downregulated after administration Q7R treatment (Figure 4). This result indicates that Q7R could effectively improve inflammation caused by the disease and alleviate the symptoms of the cholestasis. On the other hand, *FXR* mRNA expression was downregulated in the model group and upregulated after Q7R administration. These results suggest that Q7R could regulate the secretion of bile acids, maintain the mitochondrial membrane potential of hepatocytes by promoting the excretion and transport of bile acids, and play a protective role for hepatocytes.

### 3.4 Q7R administration improves inflammation and cholestasis

**FIGURE 5:**
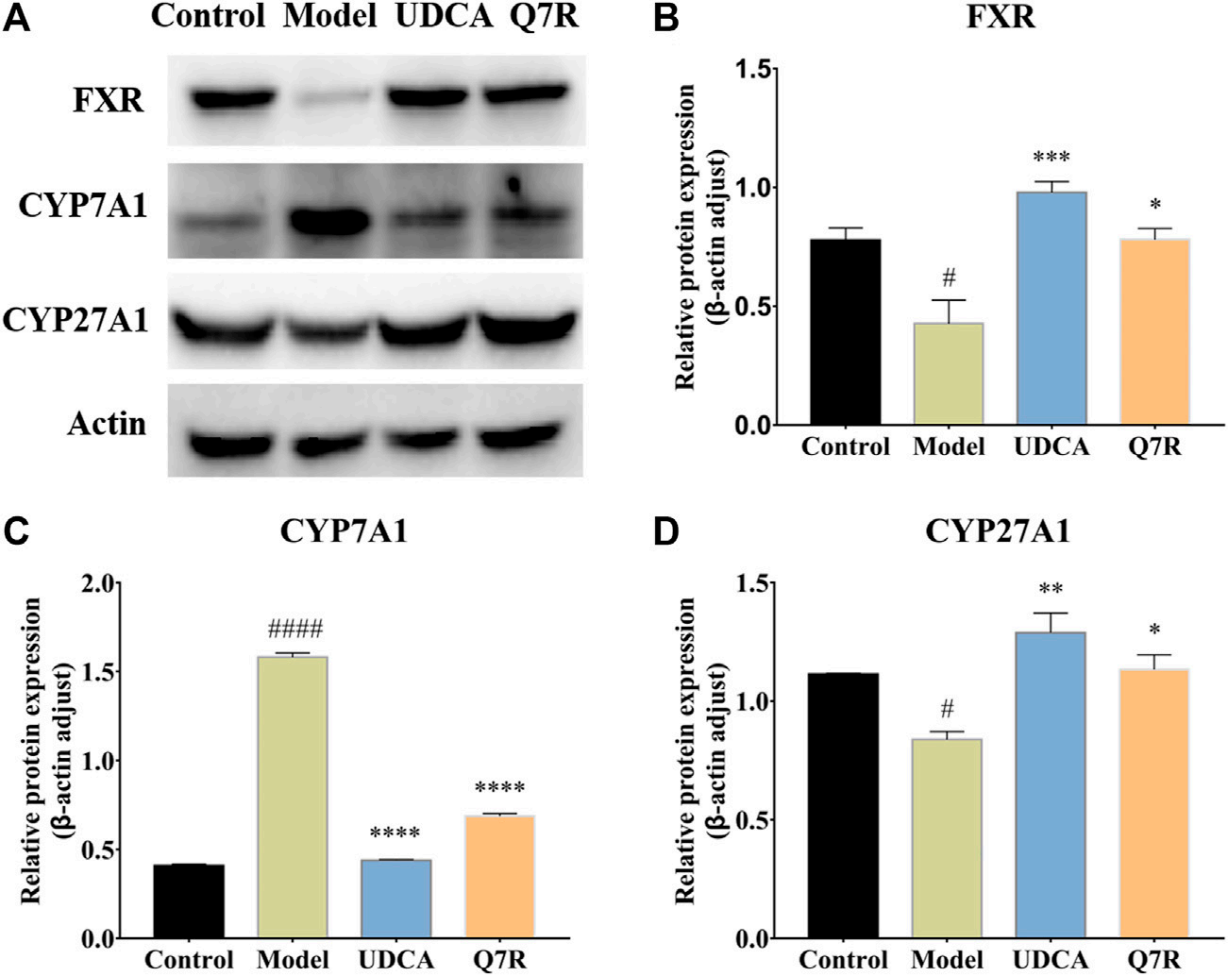
Western blot analysis for FXR, CYP7A1 and CYP27A1 protein levels in the liver of model rats (*n* = 3). **(A)** Western blot analysis of FXR, CYP7A1 and CYP27A1 protein levels; **(B)** Relative protein of FXR was quantitatively expressed by densitometric analysis, β-Actin was served as an internal control; **(C)** Relative protein of CYP7A1; **(D)** Relative protein of CYP27A1. Values are presented as mean ± SEM for three biological replicates per group. *p < .05, **p < .01, ***P <, ****p < .001 vs. the model group, ^#^ *p* < .05, ^####^ *p* < .001 vs. the control group.

To further evaluate the improvement in inflammation and cholestasis after Q7R treatment, western blotting was performed to detect the protein expression of FXR, CYP7A1, and CYP27A1. The results showed that the protein expression of FXR and CYP27A1 were downregulated in the model group, but were upregulated after Q7R administration (Figure 5), indicating that Q7R can effectively alleviate the bile acid synthesis disorder caused by the disease, alleviating the cholestasis-induced damage to hepatocytes. In addition, CYP7A1 protein expression was upregulated in the model group but downregulated after Q7R administration. This result indicates that Q7R could alleviate inflammation caused by cholestasis.

### 3.5 Q7R regulated to the bile acid metabolism

**FIGURE 6:**
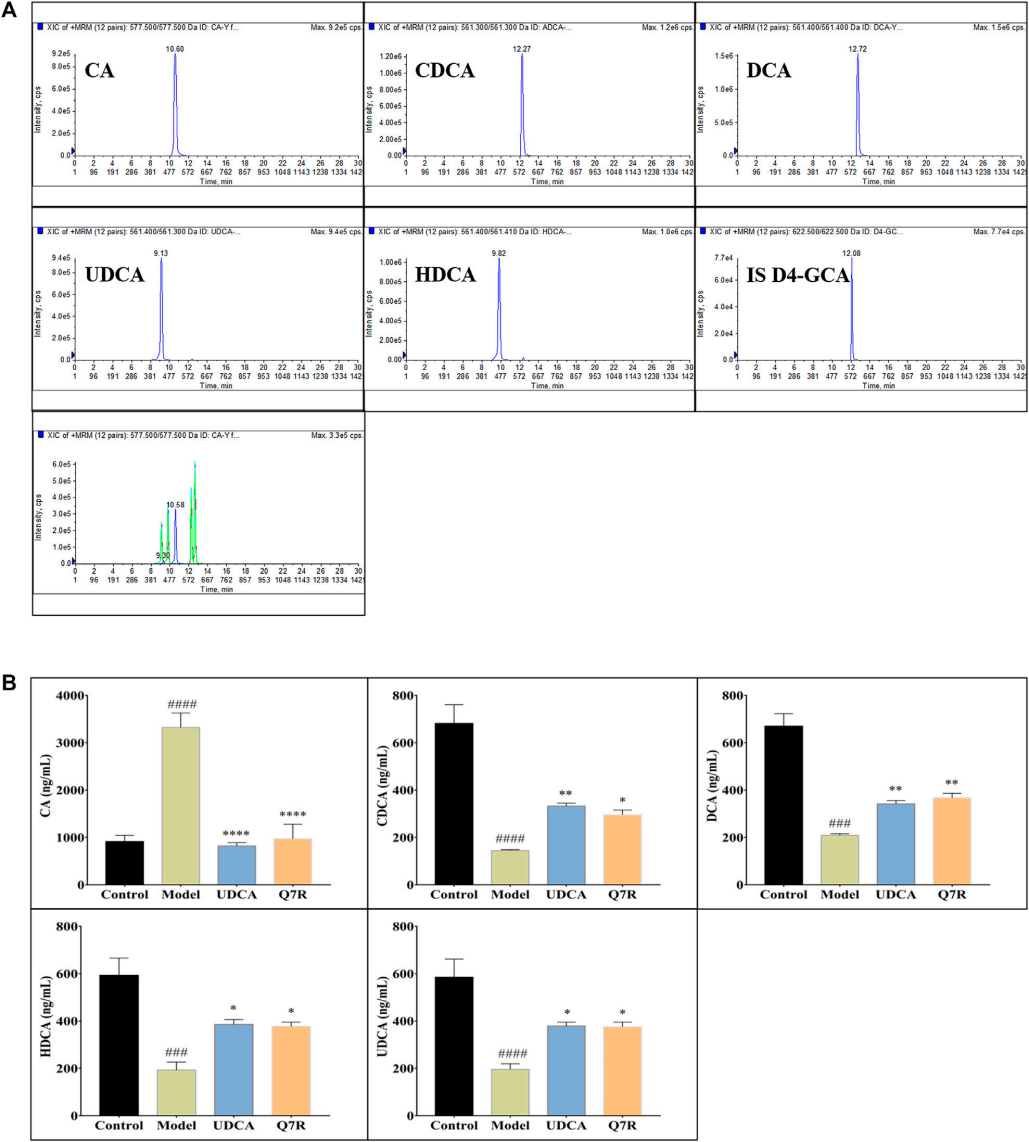
HPLC-MS/MS analysis for serum CA, CDCA, DCA, HDCA and UDCA levels in model rats (*n* = 6). **(A)** Typical chromatograms of CA, CDCA, DCA, HDCA, UDCA and internal standard (D4-GCA); **(B)** Serum CA, CDCA, DCA, HDCA and UDCA levels. Values are presented as mean ± SEM for three biological replicates per group. *p < .05, **p < .01, ****p < .001 vs. the model group, ^###^ *p* < .005, ^####^ *p* < .001 vs. the control group.

Bile acid synthesis and decomposition play a crucial role in the process of disease, the bile acid levels reflect the degree of liver injury caused by hepatotoxicity and cholestasis. In order to improve detection sensitivity of BAs and evaluate the effect of Q7R on bile acid metabolism, in this research, DIAAA was applied to establish a highly sensitive derivatization HPLC-MS/MS approach that achieved the simultaneous determination of 5 CAs in serum. The results of method validation are shown in the supplementary material (Supplementary Tables S1, S2). The correlation coefficients of the calibration curves exceeded .9990, proclaiming an excellent linearity over the concentration range employed. The accuracy varied between 95.34% and 107.30% (within-run), and 94.58% and 102.10% (between-run). The relative standard deviations (RSDs) of within- and between-run precision were below 7.47% and 7.22%, respectively. The detection results (Figure 6) showed that serum CA increased, while CDCA, DCA, UDCA and HDCA decreased in cholestatic hepatitis model rats. Notably, Q7R treatment reduced CA level, and raised the CDCA, DCA, UDCA and HDCA levels, indicating that Q7R can improve bile excretion and liver damage.

## 4 Discussion

Cholestatic hepatitis is a common liver diseas with a characterized that retention and accumulation of bile acids in the liver (Zhou et al., 2017). Many factors leading to cholestasis hepatitis disease including infections, drugs toxicity, metabolic disorder, genetic disorders, or liver transplantation. cholestatic hepatitis may progress hepatic fibrosis, cirrhosis or liver failure without appropriate treatments. Therefore, early effective intervention and potential drugs -screening for cholestatic hepatitis is extremely urgent. ANIT is a characterized cholestatic toxic agent for rats, and has widespread influence on liver basolateral and canalicular transporters. ANIT canbe conjugated with GSH in hepatocytes and transported into the bile by multidrug resistance-associated protein 2, and the ANIT-GSH conjugate dissociated into free GSH and ANIT in the bile lead to damage and necrosis of bile duct cells and liver cells. Oxidative injury and inflammation likely plays a large role in ANIT-induced cholestatic hepatitis (Wang et al., 2014).

In a sense, serum biochemical indicators reflect liver injury. The liver is the only organ that synthesizes ALB, which is required by the body and is not excreted. Therefore, the ALB level reflects the synthesis, metabolism, and reserve function of the liver. It is also an indicator to evaluate the severity and prognosis of cirrhosis, as decreased ALB levels indicate liver function damage. Liver injury leads to edema, degeneration, necrosis, and cell membrane damage in hepatocytes (Xiao et al., 2020). In this study, intracellular ALT and AST were secreted, causing serum levels to increase, reflecting the degree of liver injury (Cai et al., 2019; Liu et al., 2019). The experimental results showed that Q7R treatment could reduce ALT and AST levels and thus alleviate liver injury. Moreover, Q7R can also improve cholestasis, which can be seen from the reduction in ALT, γ-GGT, TBIL, and DBIL levels.

Bile acid is an endogenous ligand of the farnesoid X receptor (FXR), a member of the nuclear receptor (NR) superfamily, and a major regulator of bile acid biosynthesis and enterohepatic circulation. Under normal physiological conditions, liver FXR activity regulates bile acid levels while limiting bile acid accumulation (Keitel et al., 2019; Li et al., 2020). When the cholestasis occurs, the expression of FXR is inhibited; however, FXR expression was upregulated after Q7R administration. The activation of FXR inhibits the activity of CYP7A1, a key enzyme that catalyzes the *de novo* synthesis of bile acids from cholesterol. CYP7A1 inhibition reduces bile acid synthesis, bile acid accumulation in the liver tissue, and cholestasis (Zhao et al., 2017).

On the other hand, CYP27A1 is a key enzyme in the alternative pathway of bile acid synthesis. The inhibition of CYP7A1 leads to an increase in the expression of CYP27A1, which converts cholesterol into 27-hydroxycholesterol (27-HC). The molecule 27-HC is an important component of the negative feedback loop that regulates cholesterol biosynthesis. Thus, when CYP27A1 expression is elevated by pharmacological intervention, 27-HC production and cholesterol efflux are increased, and cholesterol accumulation is reduced, thereby alleviating the symptoms of the disease (Alfaqih et al., 2017).

**FIGURE 7:**
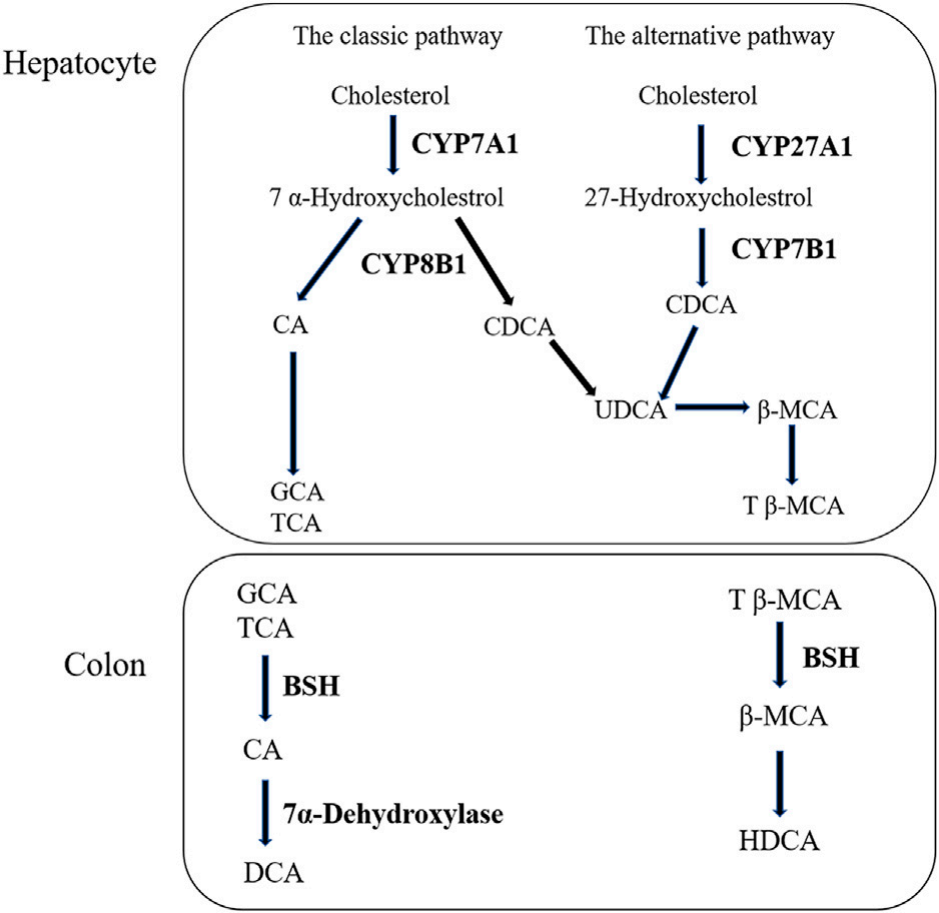
The process of bile acid metabolism.

Liver is the only organ that has all the enzymes required for bile acid synthesis. In the liver, CYP7A1 initiates the classical bile acid synthesis pathway by hydroxylating the steroid ring to form 7 α- Hydroxycholesterol. Then, CA and CDCA are generated by the catalyzed of CYP8B1, and CA continues to react to generate GCA and TCA. Next, GCA and TCA are transported out of the liver. In the intestine, GCA and TCA are hydrolyzed into CA by the bile salt hydrolase (BSH), CA at 7α- Dehydroxylase acts to biotransformed DCA. Meanwhile, in the bile acid alternative pathway, cholesterol is biotransformed to 27-HC by the action of CYP27A1 and then to CDCA by the action of CYP7B1. Similarly to the classical pathway, after a series of biotransformation, CDCA is transformed into tauro-β-Muricholic acid (T β-MCA), which is transported out of the liver and then hydrolyzed into β-MCA in the intestine. β-MCA biotransformation HDCA through 6 β-epimerization and additional 7 β-dehydroxylation (Fiorucci et al., 2004; Wahlstrom et al., 2016). According to our experimental results, the increase of CA and the decrease of CDCA are consistent with the results of protein (Tang et al., 2016). The bile acid metabolism process can be seen in Figure 7. Furthermore, DIAAA—derivatization method can improve the detection sensitivity of bile acid compounds which with carboxyl group but the disadvantage is not applicable to the all bile acid compounds. In particular, the derivative method is not suitable for taurocholic acid, tauroursodeoxycholic acid, taurochenodeoxycholic acid et al., which the chemical structure with sulfonyl group. It is necessary to cooperate with other detection methods to detect all bile acids, we will continue to explore the most suitable detection technology in future research.

An inflammatory response can be induced in the early stage of cholestasis, producing many inflammatory mediators, such as cytokines and chemokines, which induce the activation of neutrophils and their infiltration and aggregation in liver tissue (Liew and Kubes, 2019). Bile acid-induced liver inflammation induces pro-inflammatory cytokines to activate NF-κB (Liu et al., 2017), which inhibits the transcription of FXR in the liver and prevents the subsequent activation of the small heterodimer chaperone (SHP). SHP activation inhibits the synthesis of the bile-producing rate-limiting enzyme CYP7A1 *via* the activated SHP pathway (Shaik et al., 2015). Furthermore, NCAO2, also known as steroid receptor coactivator 2 (SRC2), is a member of the p160 steroid receptor coactivator family (Suresh et al., 2017), whose target gene is SHP. Therefore, the role of NCAO2 in liver bile acid homeostasis has been widely studied.

On the other hand, TNF-α is a pro-inflammatory cytokine involved in normal inflammatory and immune responses and is mainly produced by activated monocytes and macrophages (Idriss and Naismith, 2000). TNF-α production is thought to occur primarily through signals that activate NF-κB. IL-1β is another pro-inflammatory cytokine that plays a key role in acute and chronic inflammation. Inflammasomes can promote the maturation of IL-1β (Kaneko et al., 2019). As a target gene of NF-κB, it regulates the recruitment and activation of other immune cells after activation to amplify the inflammatory response.

PTGS2, also known as COX2, is an enzyme capable of initiating inflammation and promoting prostaglandin synthesis upon stimulation by various inflammatory factors, such as cytokines and bacteria. Prostaglandins can alter hepatic bile flow and may be related to the release and action of some cholelithogenic hormones. It is also involved in gallbladder contraction and water absorption and is closely related to the occurrence of cholecystitis and gallstones (Wang et al., 2021). Similarly, PTGS1 is an enzyme that constitutes prostaglandins, and its expression is closely related to inflammation.

## 5 Conclusion

In conclusion, this study demonstrated that Q7R could activate FXR, CYP7A1, and CYP27A1, effectively regulate bile acid synthesis disorders, and ameliorate hepatocyte membrane damage. Moreover, Q7R can also reduce TNF-α, IL-1β, PTGS1, PTGS2, NCOA2, and NF-κB levels and alleviate the inflammatory response caused by cholestasis. Additionally, Q7R plays a hepatoprotective role. Taken together, the results of this study may provide support for further research and clinical application of Q7R in cholestatic hepatitis.

## Data Availability

The original contributions presented in the study are included in the article/Supplementary Material, further inquiries can be directed to the corresponding authors.
