# Supplementary material for: Quercetin 7-rhamnoside protects against alpha-naphthylisothiocyanate (ANIT)-induced in cholestatic hepatitis rats by improving biliary excretion and inhibiting inflammatory responses
Source: Front Pharmacol. 2023 Jan 9;13:1116257. doi: 10.3389/fphar.2022.1116257 (PMC9868710; doi:10.3389/fphar.2022.1116257)
Supplement: Supplementary file 1 [file DataSheet1.ZIP › Supplementary material-The original results of Methodological evaluation and Western blot and figure legend--Revised version/Supplementary Material.docx]

Supplementary Material

# Supplementary Data

**1.1 Methodological evaluation**

Supplementary Table 1. Calibration curves in 3 different days in 5% BSA.

| Cpd. |  | Standard curves | R^2^ | Ranger |
| --- | --- | --- | --- | --- |
|  | Day 1 | *y = 0.0148 x + 0.2836* | 0.9995 |  |
| CA | Day 2 | *y = 0.0234 x + 0.2094* | 0.9990 | 200-20000 ng/ml |
|  | Day 3 | *y = 0.0023 x + 0.2004* | 0.9991 |  |
|  | Day 1 | *y = 0.0133 x + 0.4136* | 0.9994 |  |
| CDCA | Day 2 | *y = 0.0464 x + 0.4195* | 0.9991 | 200-20000 ng/ml |
|  | Day 3 | *y = 0.01 x + 0.4261* | 0.9992 |  |
|  | Day 1 | *y = 0.0145 x + 0.4203* | 0.9991 |  |
| DCA | Day 2 | *y = 0.0147 x + 0.4568* | 0.9994 | 200-20000 ng/ml |
|  | Day 3 | *y = 0.0191 x + 0.4943* | 0.9995 |  |
|  | Day 1 | *y = 0.0118 x + 0.4016* | 0.9991 |  |
| UDCA | Day 2 | *y = 0.0054 x + 0.4088* | 0.9993 | 200-20000 ng/ml |
|  | Day 3 | *y = 0.0067 x + 0.4144* | 0.9997 |  |
|  | Day 1 | *y = 0.0343 x + 0.4551* | 0.9992 |  |
| HDCA | Day 2 | *y = 0.0377 x + 0.4999* | 0.9992 | 200-20000 ng/ml |
|  | Day 3 | *y = 0.0052 x + 0.4451* | 0.9992 |  |

Supplementary Table 2. Within-run and between-run precision and accuracy values in 5% BSA (n = 3).

|  |  | Intra-day | | Inter-day | |
| --- | --- | --- | --- | --- | --- |
|  | c(ng/mL) | Accuracy (%) | Precision (%) | Accuracy (%) | Precision (%) |
| CA | 500 | 102.16±5.00 | 2.27 | 98.08±6.04 | 6.16 |
|  | 2000 | 102.17±5.29 | 2.33 | 99.63±7.20 | 7.22 |
|  | 10000 | 105.03±5.67 | 5.46 | 100.47±1.98 | 1.97 |
| CDCA | 500 | 96.24±2.18 | 5.36 | 95.38±3.26 | 3.42 |
|  | 2000 | 97.84±2.28 | 5.94 | 95.44±5.31 | 5.56 |
|  | 10000 | 95.34±5.21 | 7.47 | 98.42±6.99 | 7.11 |
| DCA | 500 | 100.33±5.52 | 5.50 | 94.58±1.72 | 1.81 |
|  | 2000 | 98.24±3.38 | 3.44 | 94.94±6.11 | 6.44 |
|  | 10000 | 99.11±6.70 | 6.76 | 96.38±2.71 | 2.81 |
| UDCA | 500 | 103.52±4.91 | 4.75 | 98.04±6.36 | 6.49 |
|  | 2000 | 107.30±2.25 | 2.09 | 102.10±4.20 | 4.11 |
|  | 10000 | 102.50±6.04 | 5.89 | 96.79±1.06 | 1.09 |
| HDCA | 500 | 95.78±4.24 | 4.43 | 95.45±3.70 | 3.87 |
|  | 2000 | 102.57±1.19 | 1.16 | 101.48±2.47 | 2.44 |
|  | 10000 | 95.69±4.86 | 5.08 | 93.66±1.32 | 1.41 |

# Supplementary Figures

# 2.1 Figure legends of supplementary material


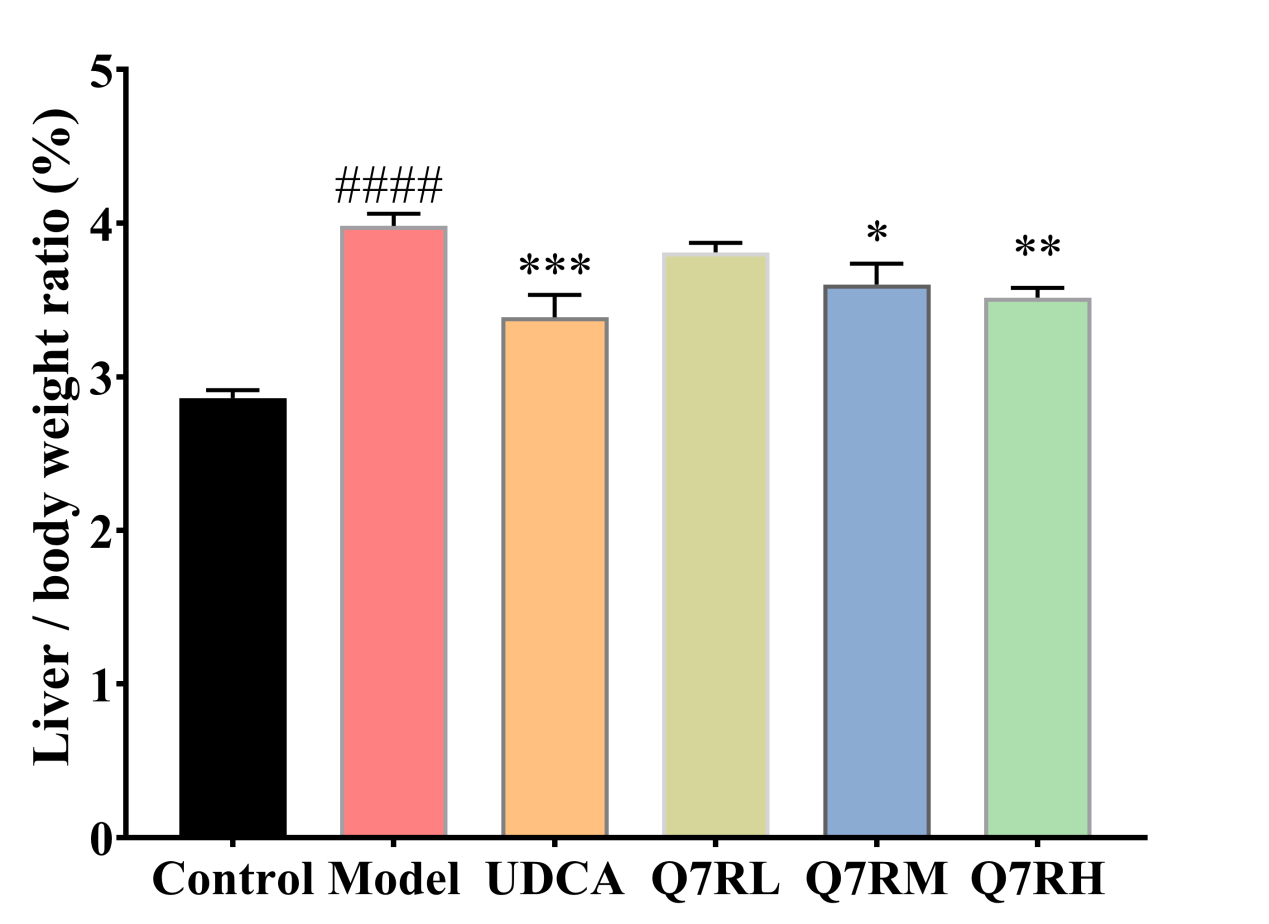


**Figure S1** Area of collage fibers in liver according to masson stain. *** *P* < 0.005 vs. model, ^###^ *P* < 0.005 vs. control. Q7R, Quercetin 7-rhamnoside.


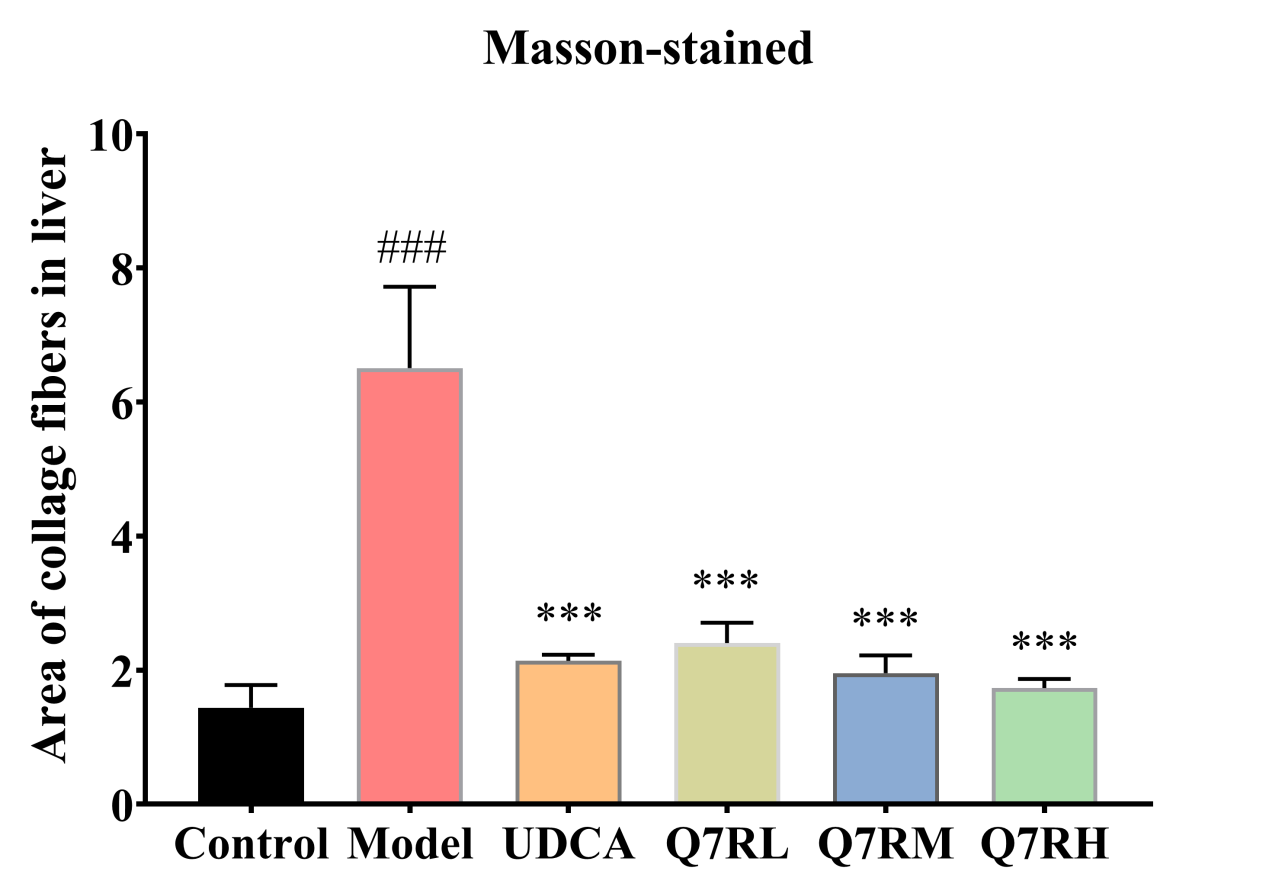


**Figure S2** Effect of Q7R treatment on the liver index in the ANIT rats (n=6). * *P* < 0.05, ** *P* < 0.01 vs. model, ^####^ *P* < 0.001 vs. control.

**Figure S3** Western Blot analysis for FXR protein levels in the liver of model rats. (S1-1). Actin in **Figure 5A**. (S1-2) Actin of FXR-2. (S1-3) Actin of FXR-3. (S1-4) FXR in **Figure 5A**. (S1-5) FXR-2. (S1-6) FXR-3.

**Figure S4** Western blot analysis for CYP7A1 protein levels in the liver of model rats. (S2-1). Actin of CYP7A1. (S2-2). Actin of CYP7A1-2. (S2-3). Actin of CYP7A1-3. (S2-4). CYP7A1 in **Figure 5A**. (S2-5). CYP7A1-2. (S2-6). CYP7A1-3.

**Figure S5** Western blot analysis for CYP27A1 protein levels in the liver of model rats. (S3-1). Actin of CYP27A1. (S3-2). Actin of CYP27A1-2. (S3-3). Actin of CYP27A1-3. (S3-4). CYP27A1 in **Figure 5A**. (S3-5). CYP27A1-2. (S3-6). CYP27A1-3.
