# Supplementary figures and images for: Quercetin 7-rhamnoside protects against alpha-naphthylisothiocyanate (ANIT)-induced in cholestatic hepatitis rats by improving biliary excretion and inhibiting inflammatory responses
Source: Front Pharmacol. 2023 Jan 9;13:1116257. doi: 10.3389/fphar.2022.1116257 (PMC9868710; doi:10.3389/fphar.2022.1116257)

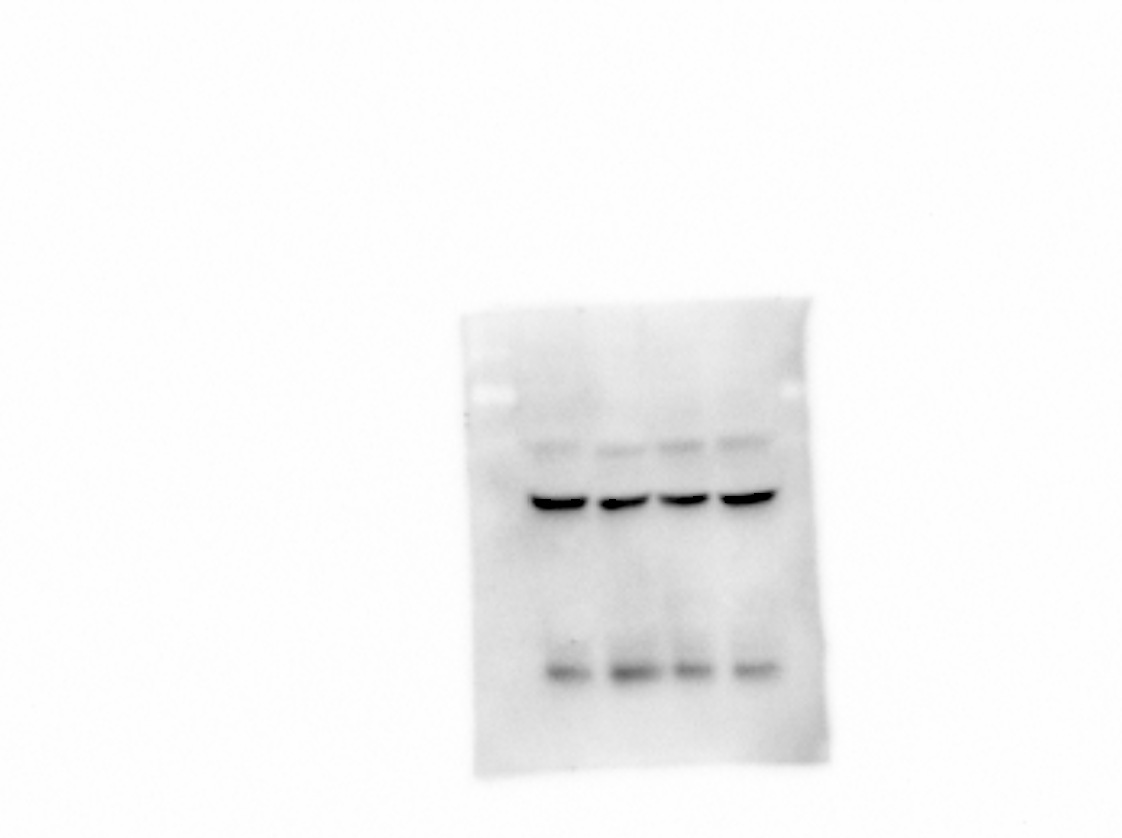

Supplement: Supplementary file 1 [file DataSheet1.ZIP › Supplementary material-The original results of Methodological evaluation and Western blot and figure legend--Revised version/Figure 5 A/S1-1 Actin in Figure 5A.tif]

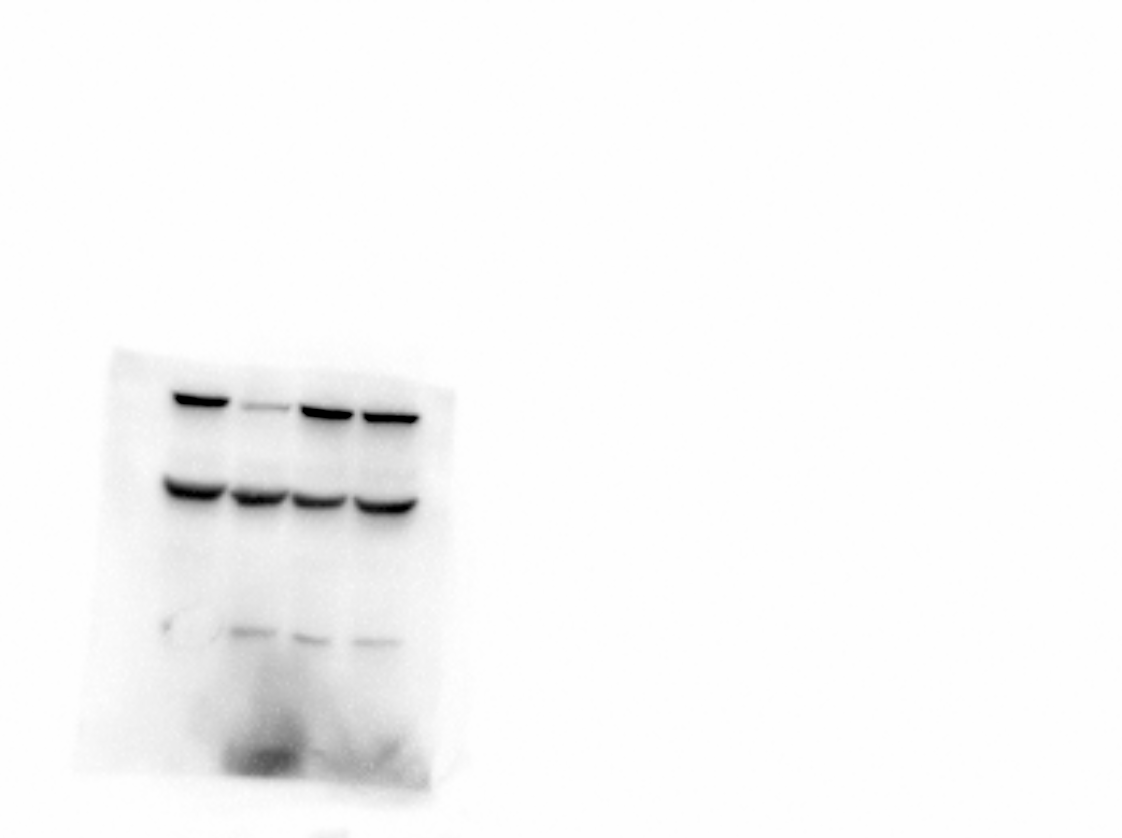

Supplement: Supplementary file 1 [file DataSheet1.ZIP › Supplementary material-The original results of Methodological evaluation and Western blot and figure legend--Revised version/Figure 5 A/S1-4 FXR in Figure 5A.tif]

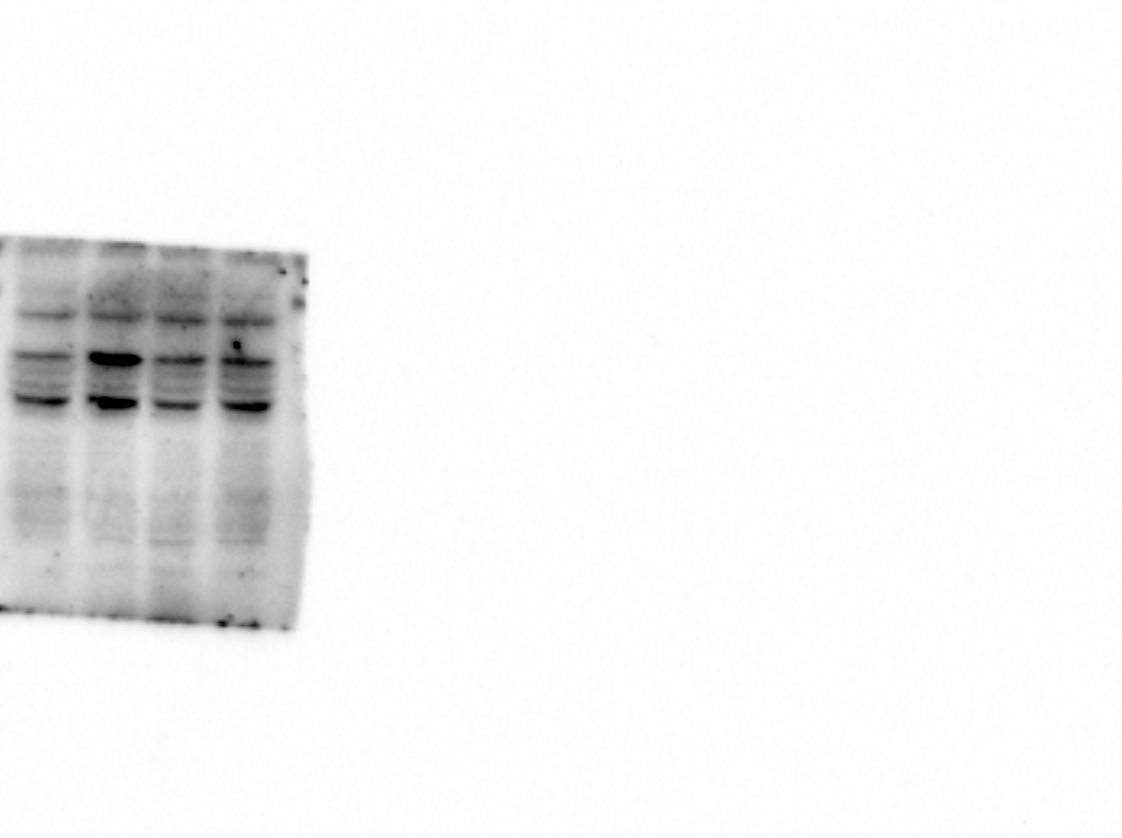

Supplement: Supplementary file 1 [file DataSheet1.ZIP › Supplementary material-The original results of Methodological evaluation and Western blot and figure legend--Revised version/Figure 5 A/S2-4 CYP7A1 in Figure 5A.tif]

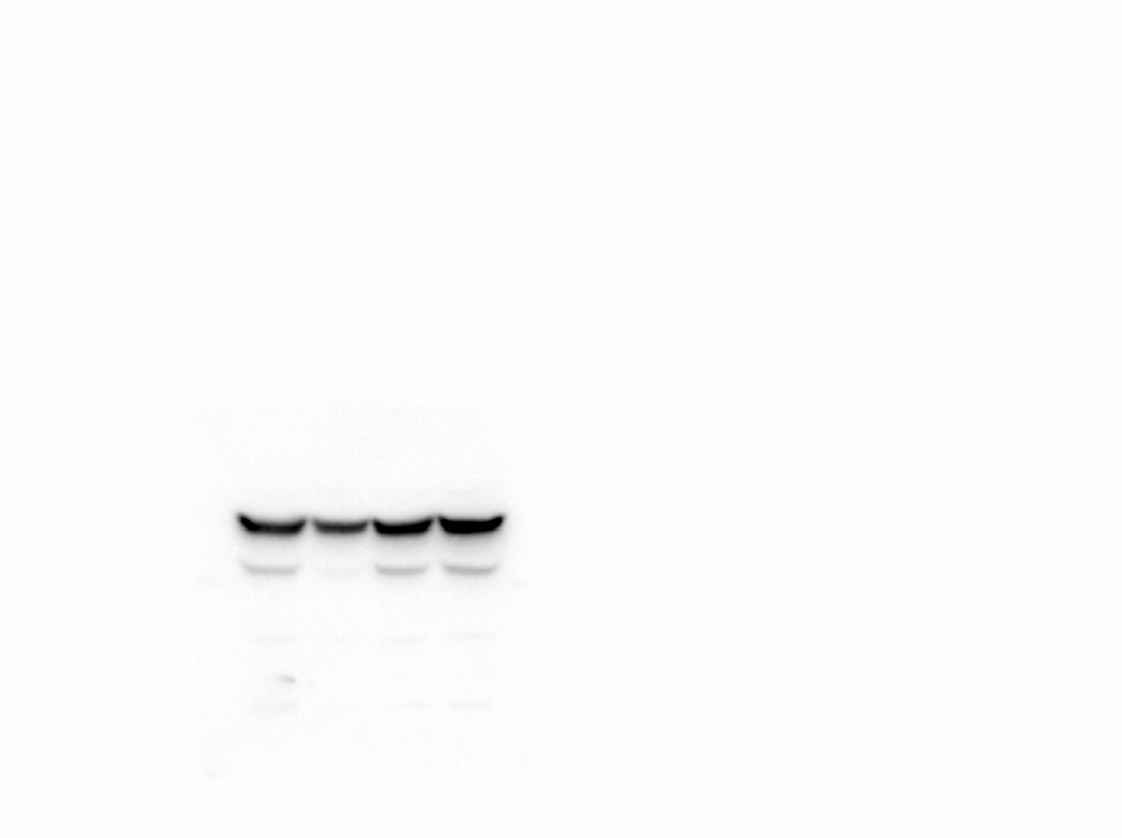

Supplement: Supplementary file 1 [file DataSheet1.ZIP › Supplementary material-The original results of Methodological evaluation and Western blot and figure legend--Revised version/Figure 5 A/S3-4 CYP27A1 in Figure 5A.tif]

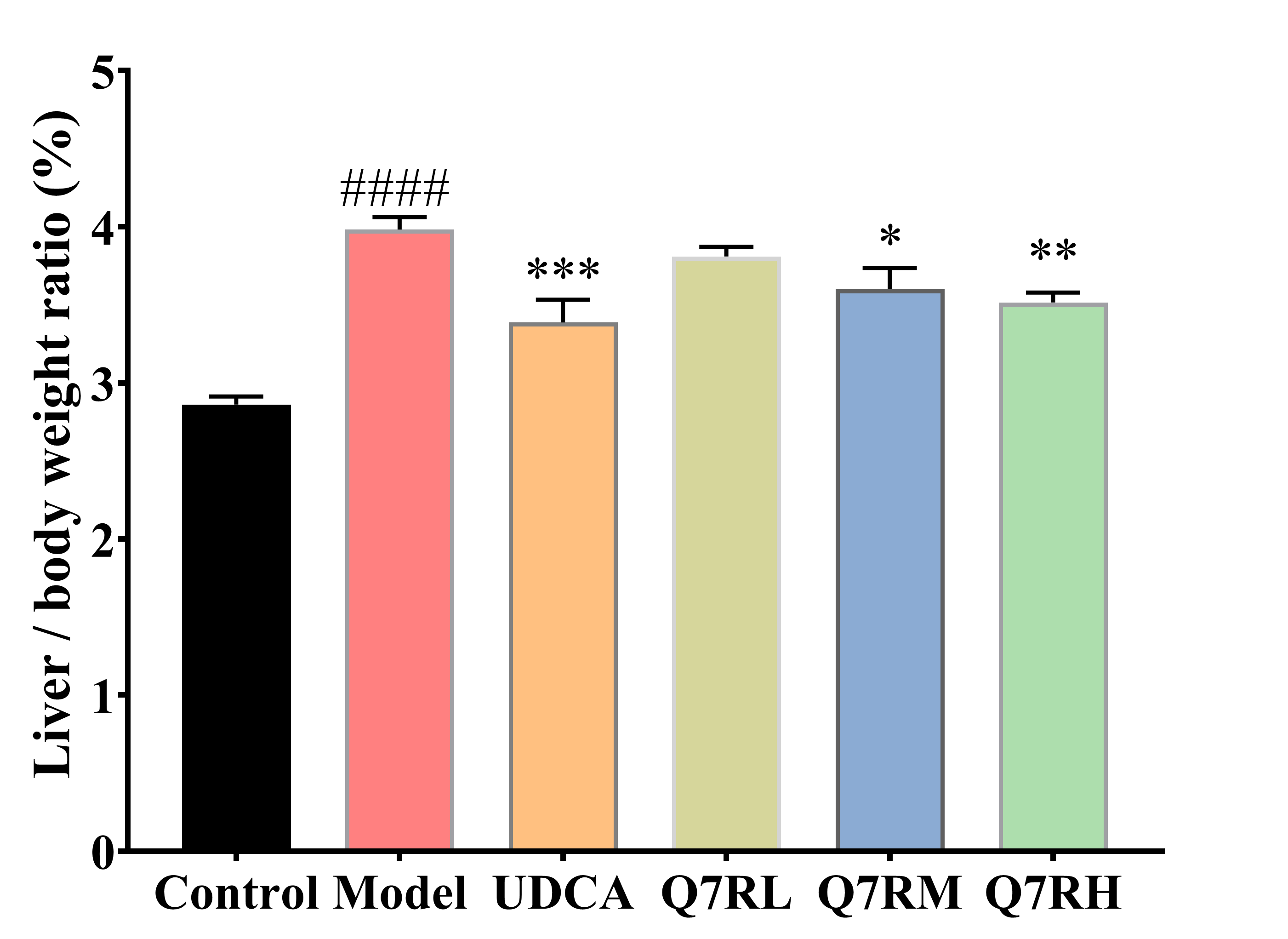

Supplement: Supplementary file 1 [file DataSheet1.ZIP › Supplementary material-The original results of Methodological evaluation and Western blot and figure legend--Revised version/Figure S1 Liver index.tif]

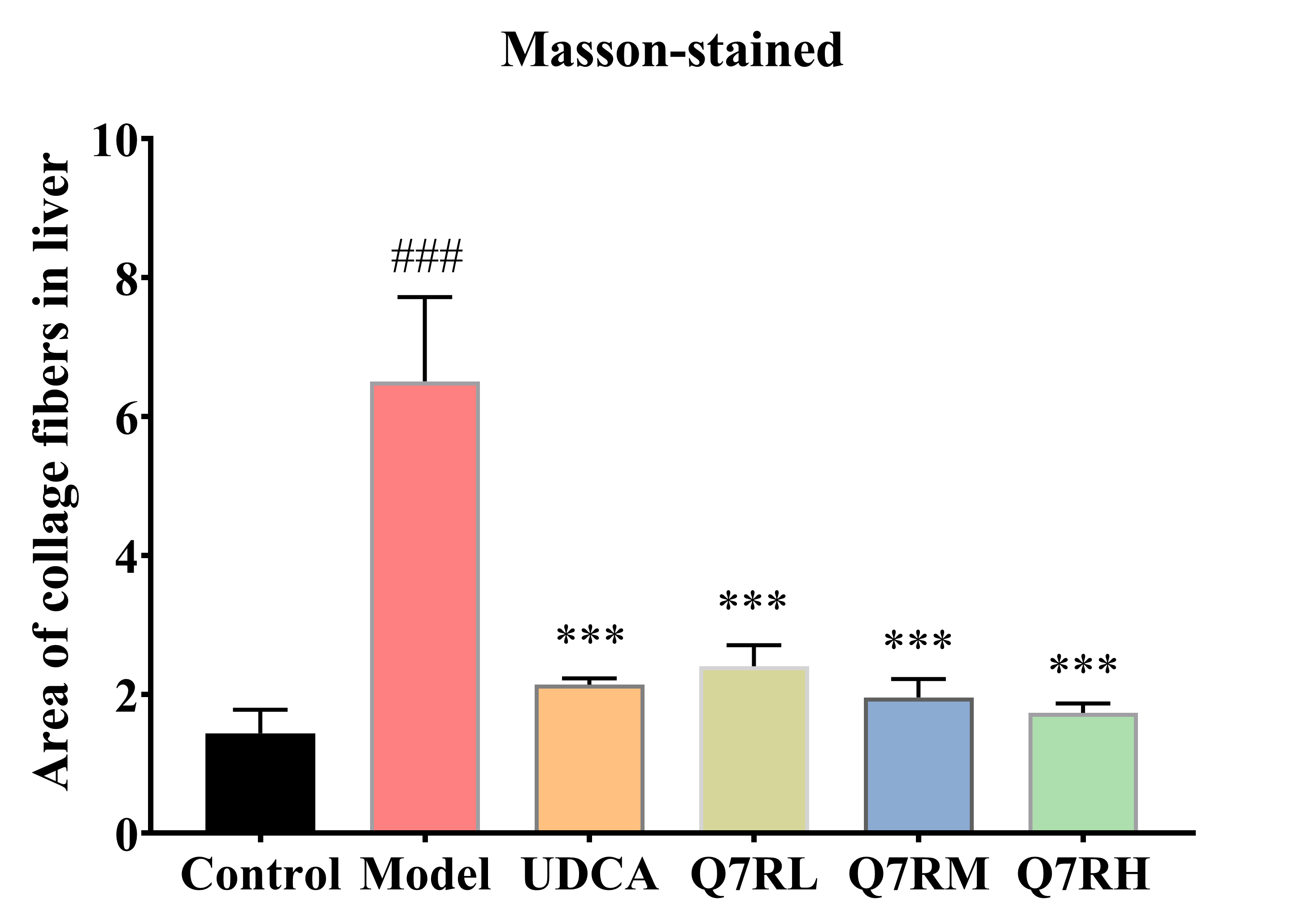

Supplement: Supplementary file 1 [file DataSheet1.ZIP › Supplementary material-The original results of Methodological evaluation and Western blot and figure legend--Revised version/Figure S2 Masson Stain.tif]

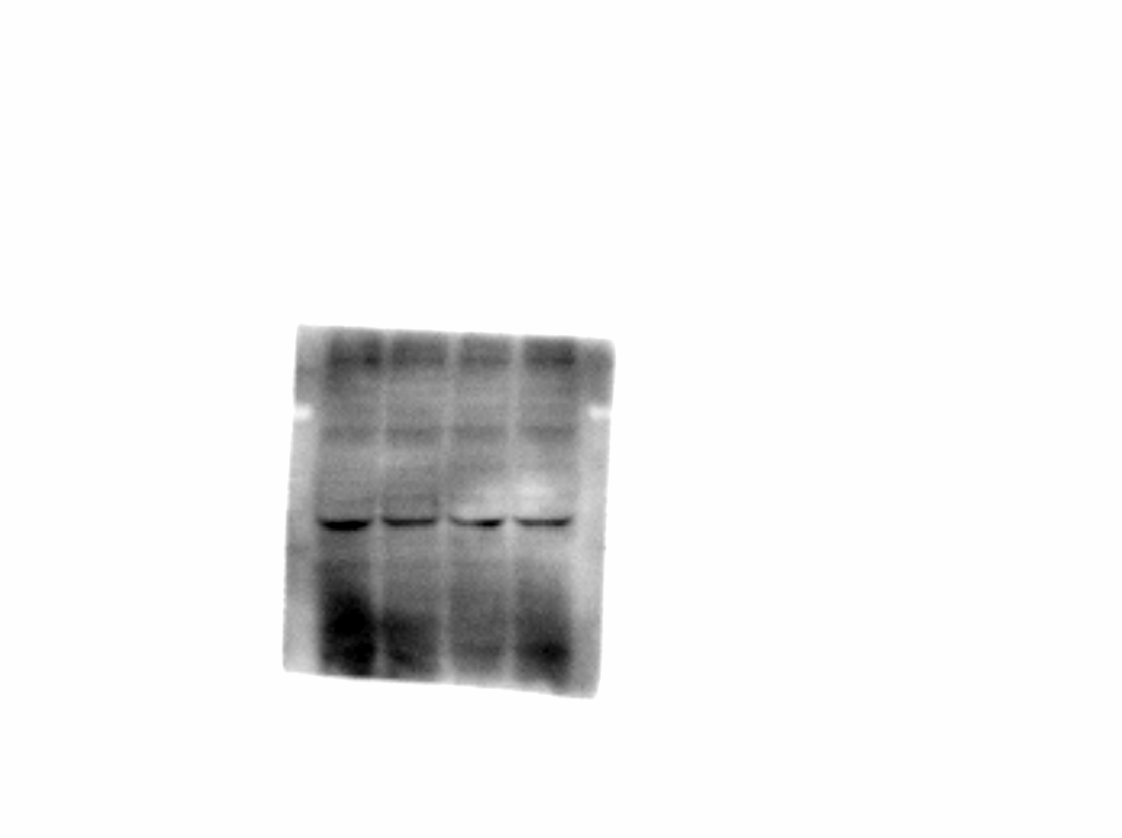

Supplement: Supplementary file 1 [file DataSheet1.ZIP › Supplementary material-The original results of Methodological evaluation and Western blot and figure legend--Revised version/Replication experiment/S1-2 Actin of FXR-2.tif]

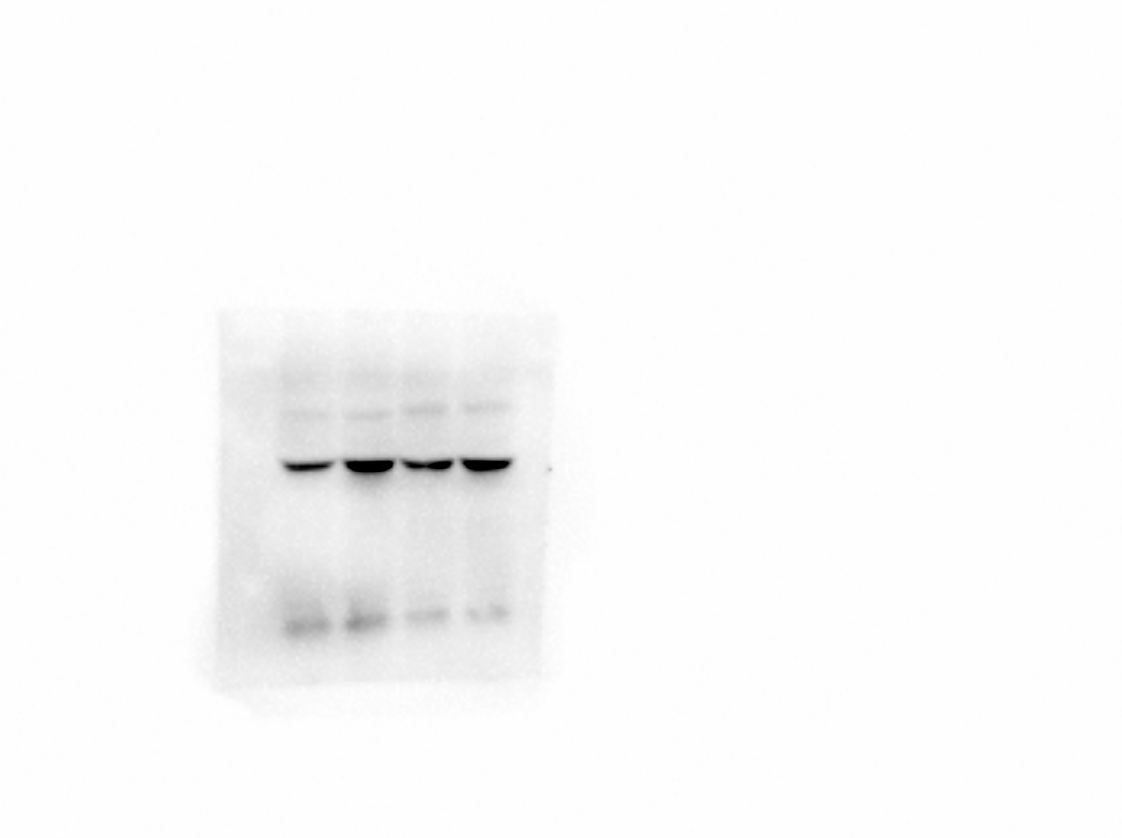

Supplement: Supplementary file 1 [file DataSheet1.ZIP › Supplementary material-The original results of Methodological evaluation and Western blot and figure legend--Revised version/Replication experiment/S1-3 Actin of FXR-3.tif]

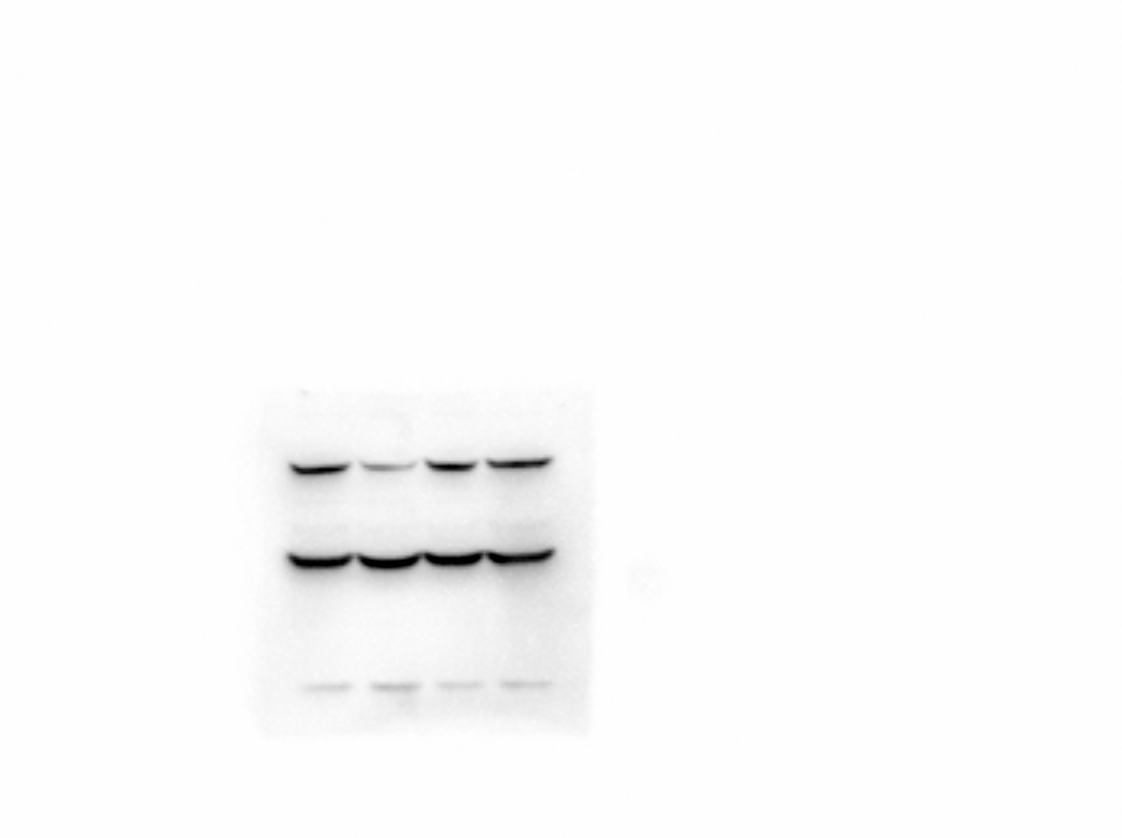

Supplement: Supplementary file 1 [file DataSheet1.ZIP › Supplementary material-The original results of Methodological evaluation and Western blot and figure legend--Revised version/Replication experiment/S1-5 FXR-2.tif]

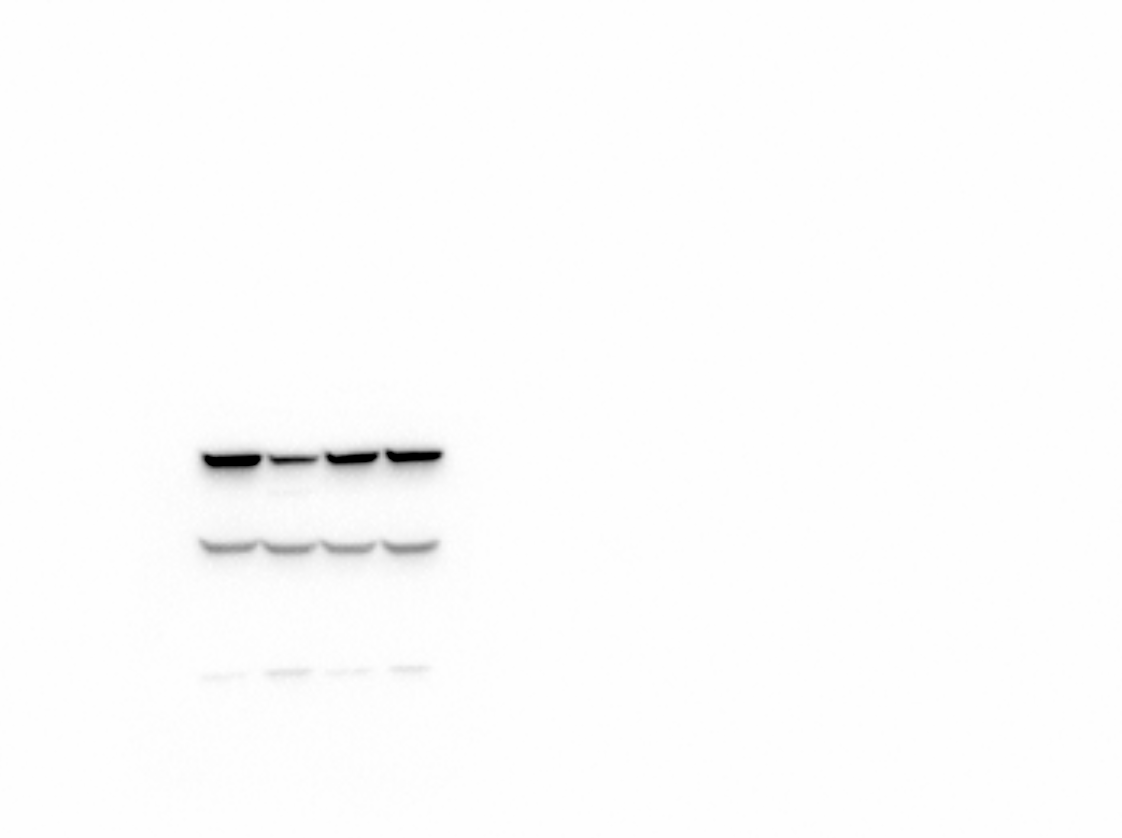

Supplement: Supplementary file 1 [file DataSheet1.ZIP › Supplementary material-The original results of Methodological evaluation and Western blot and figure legend--Revised version/Replication experiment/S1-6 FXR-3.tif]

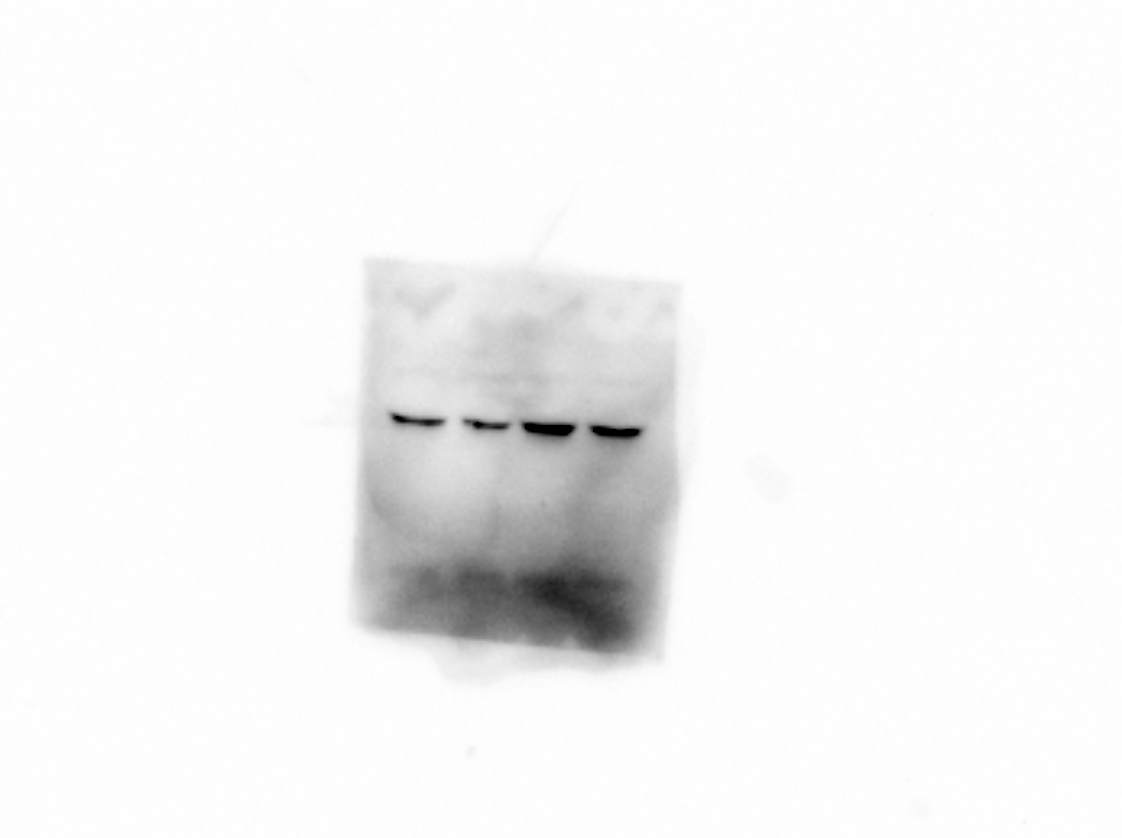

Supplement: Supplementary file 1 [file DataSheet1.ZIP › Supplementary material-The original results of Methodological evaluation and Western blot and figure legend--Revised version/Replication experiment/S2-1 Actin of CYP7A1.tif]

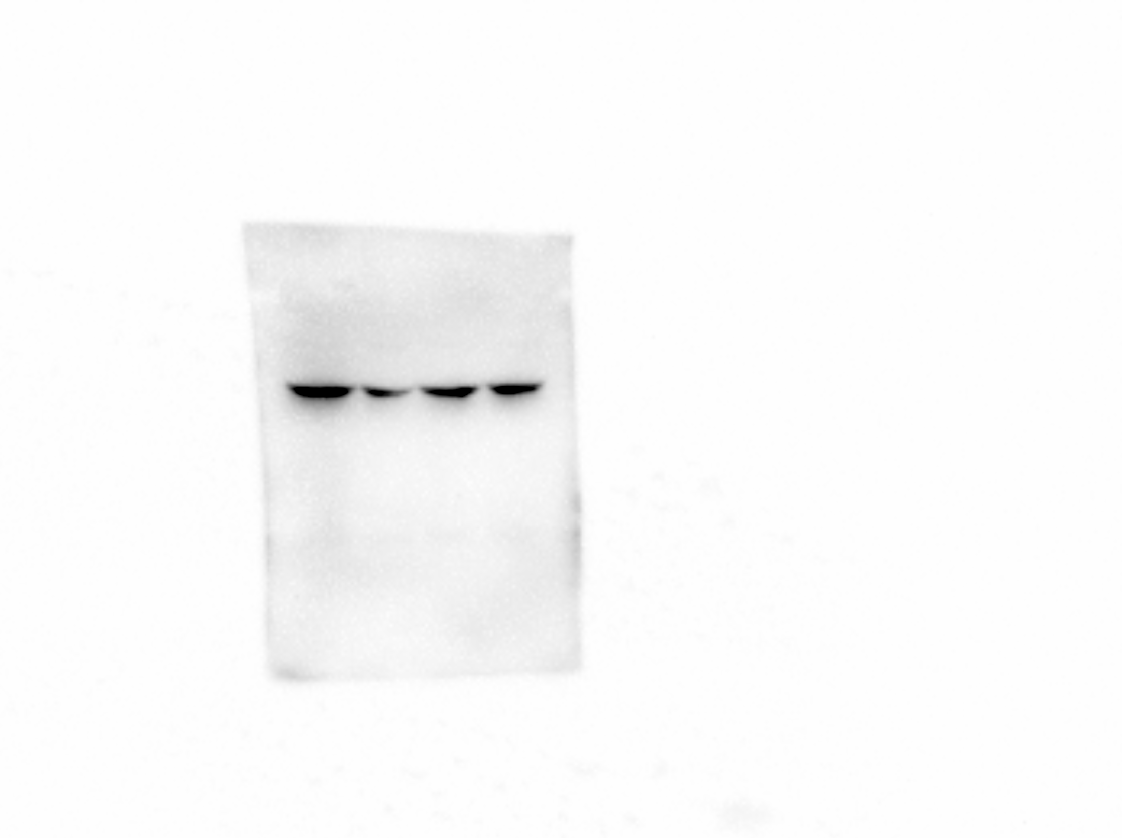

Supplement: Supplementary file 1 [file DataSheet1.ZIP › Supplementary material-The original results of Methodological evaluation and Western blot and figure legend--Revised version/Replication experiment/S2-2 Actin of CYP7A1-2.tif]

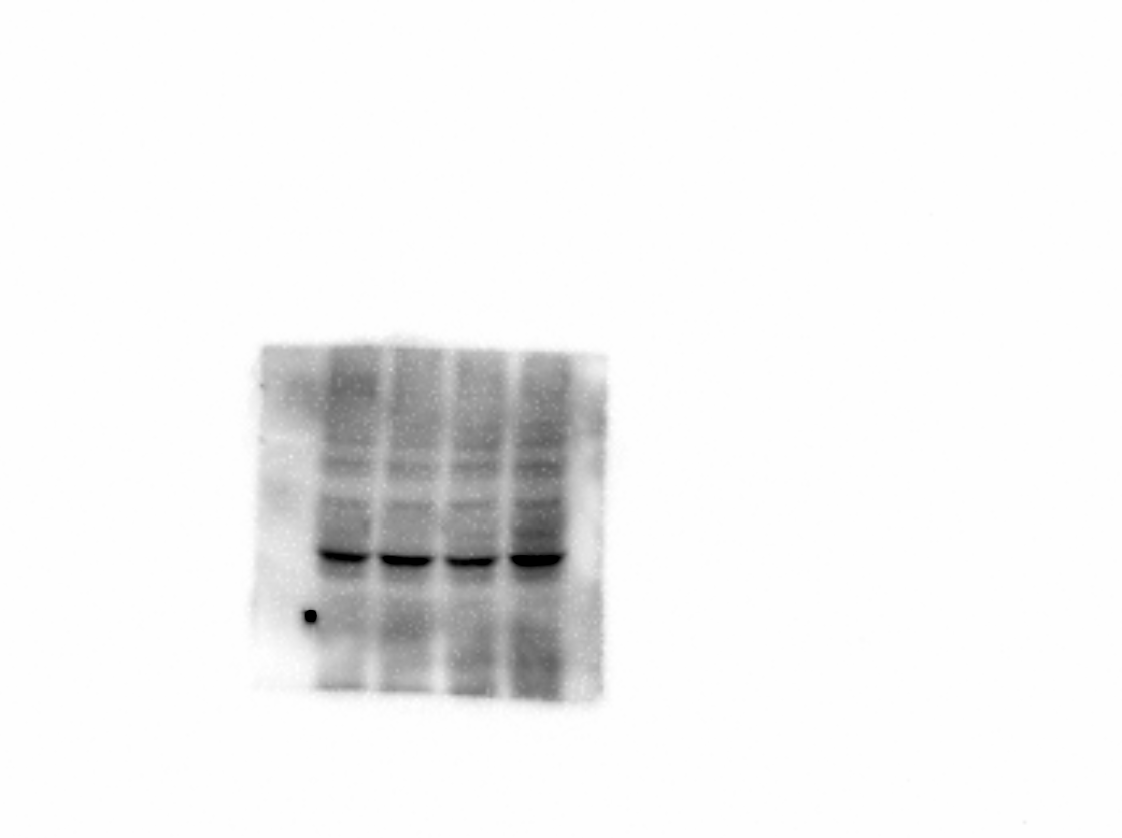

Supplement: Supplementary file 1 [file DataSheet1.ZIP › Supplementary material-The original results of Methodological evaluation and Western blot and figure legend--Revised version/Replication experiment/S2-3 Actin of CYP7A1-3.tif]

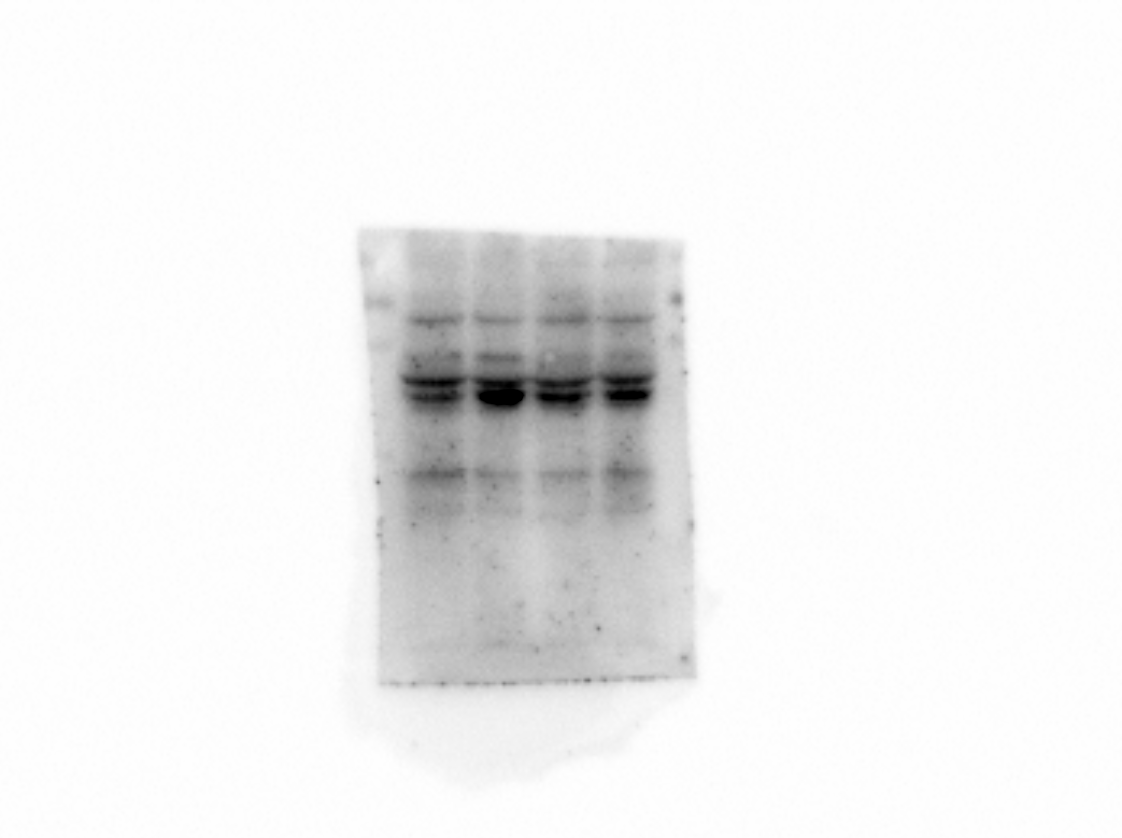

Supplement: Supplementary file 1 [file DataSheet1.ZIP › Supplementary material-The original results of Methodological evaluation and Western blot and figure legend--Revised version/Replication experiment/S2-5 CYP7A1-2.tif]

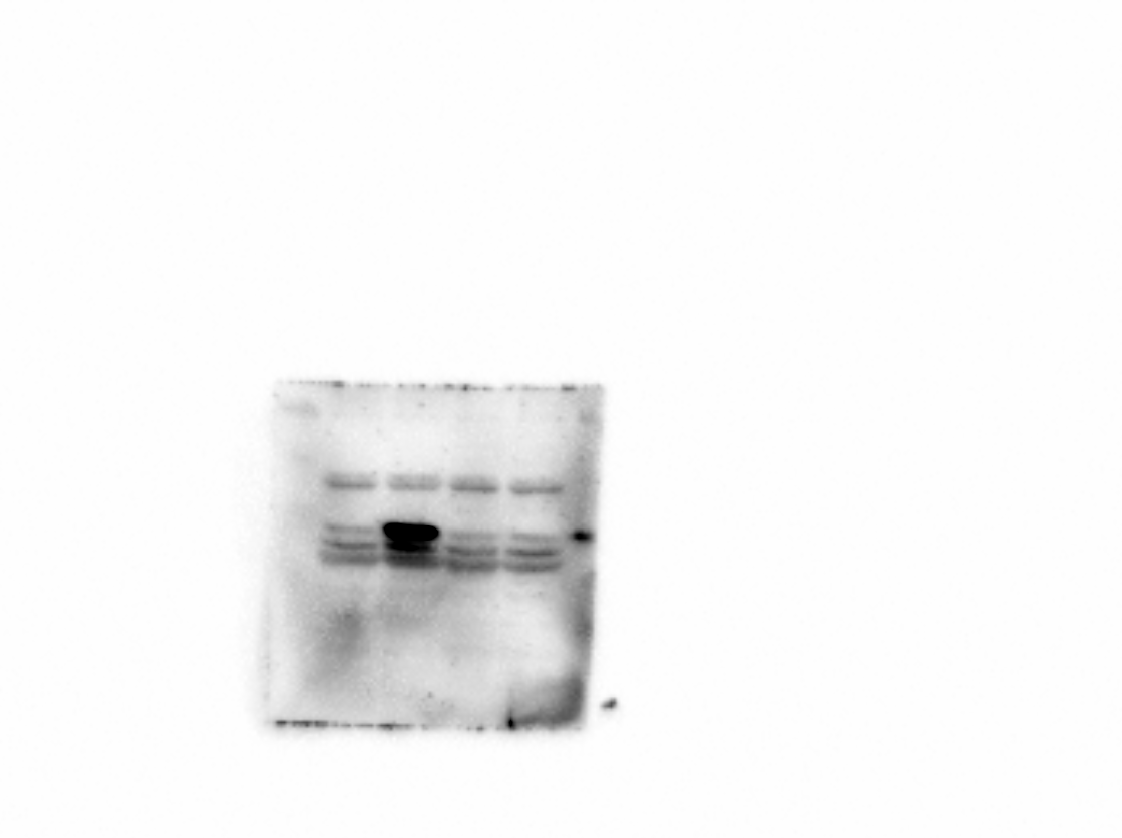

Supplement: Supplementary file 1 [file DataSheet1.ZIP › Supplementary material-The original results of Methodological evaluation and Western blot and figure legend--Revised version/Replication experiment/S2-6 CYP7A1-3.tif]

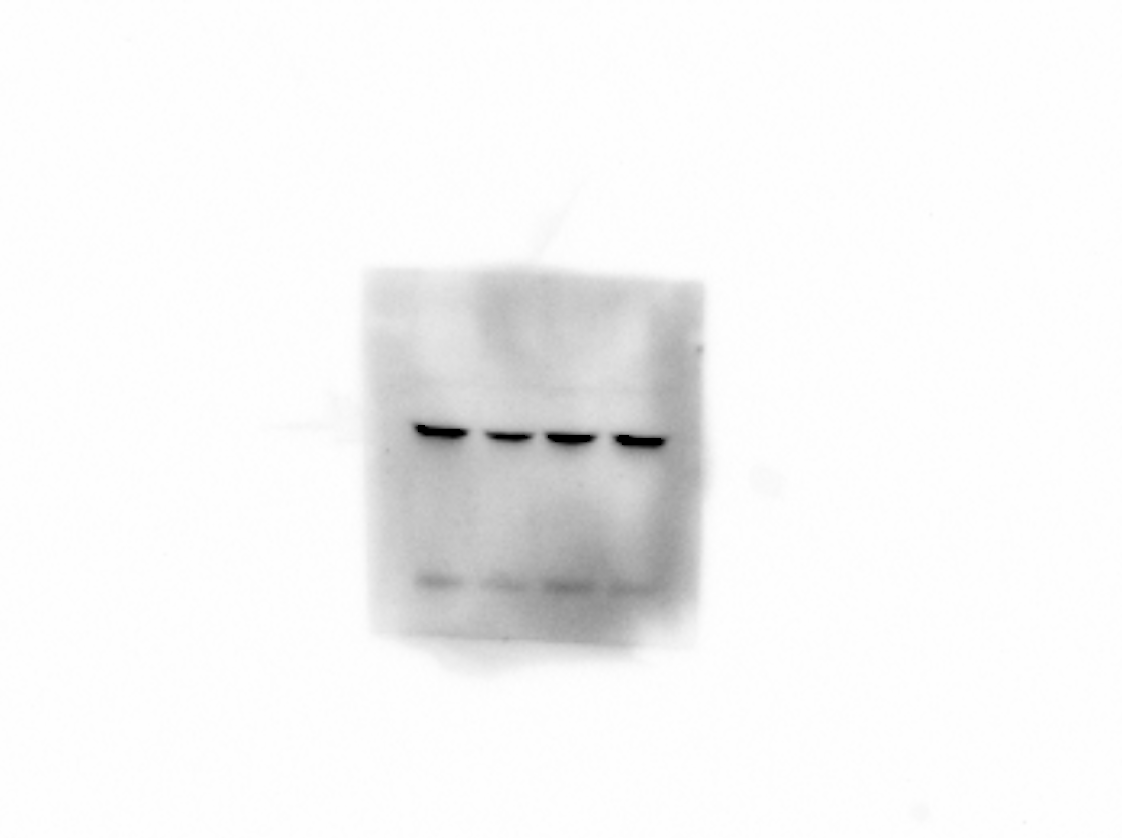

Supplement: Supplementary file 1 [file DataSheet1.ZIP › Supplementary material-The original results of Methodological evaluation and Western blot and figure legend--Revised version/Replication experiment/S3-1 Actin of CYP27A1.tif]

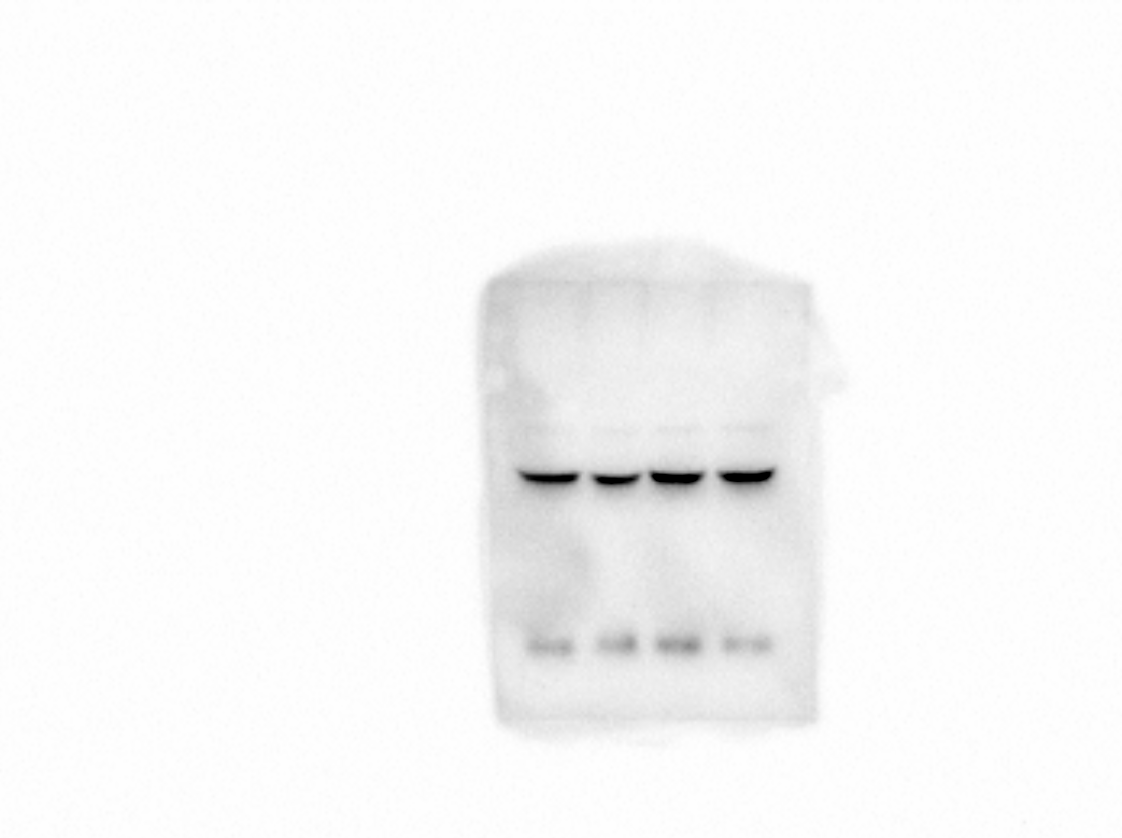

Supplement: Supplementary file 1 [file DataSheet1.ZIP › Supplementary material-The original results of Methodological evaluation and Western blot and figure legend--Revised version/Replication experiment/S3-2 Actin of CYP27A1-2.tif]

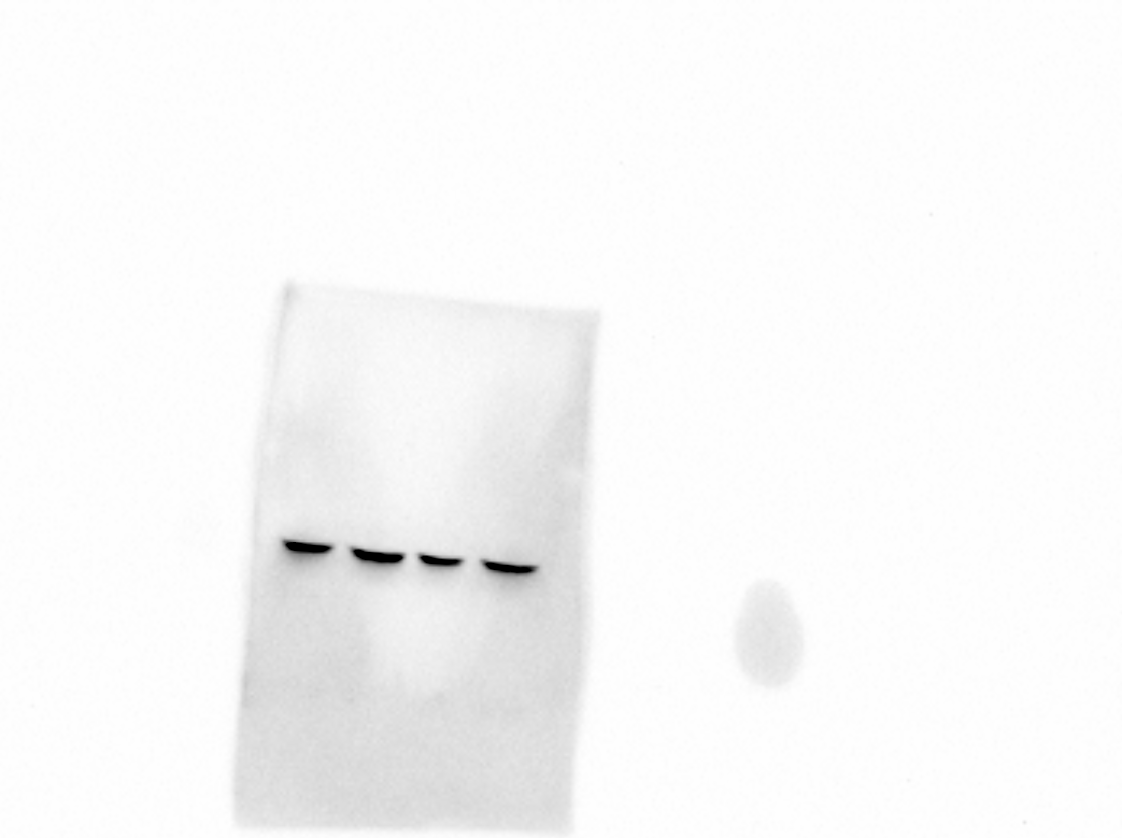

Supplement: Supplementary file 1 [file DataSheet1.ZIP › Supplementary material-The original results of Methodological evaluation and Western blot and figure legend--Revised version/Replication experiment/S3-3 Actin of CYP27A1-3.tif]

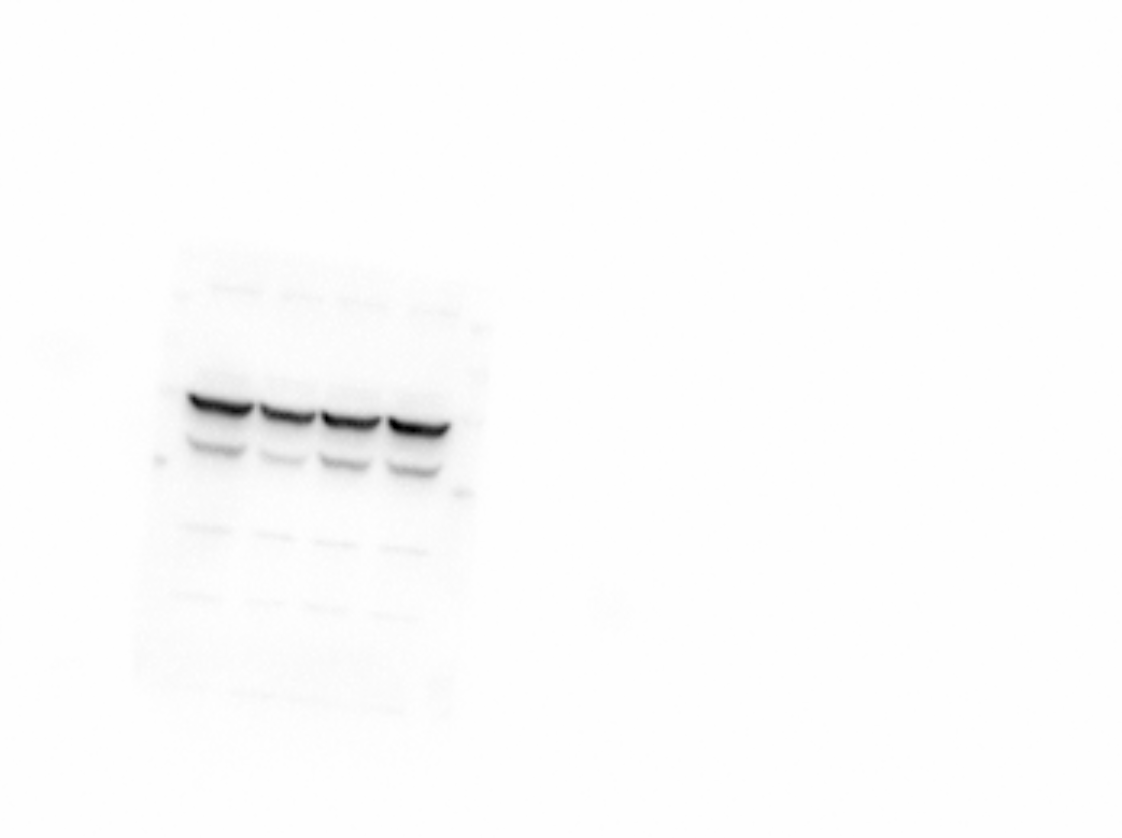

Supplement: Supplementary file 1 [file DataSheet1.ZIP › Supplementary material-The original results of Methodological evaluation and Western blot and figure legend--Revised version/Replication experiment/S3-5 CYP27A1-2.tif]

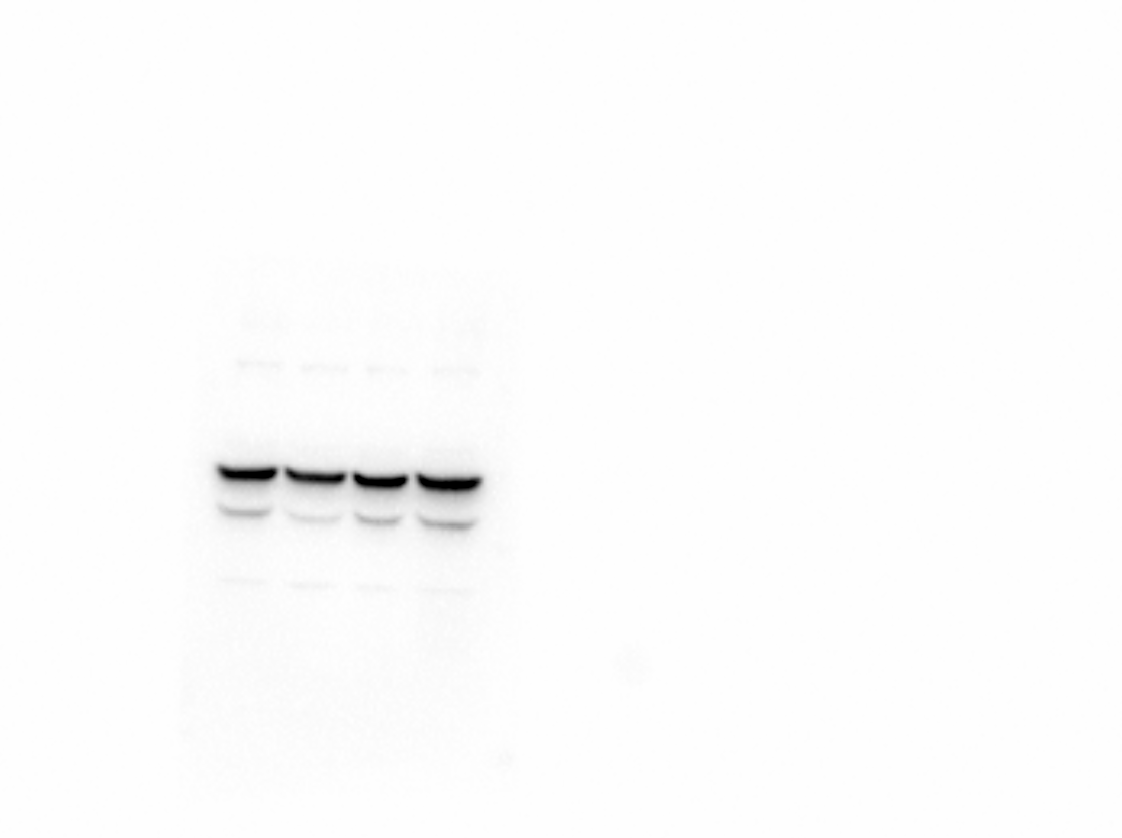

Supplement: Supplementary file 1 [file DataSheet1.ZIP › Supplementary material-The original results of Methodological evaluation and Western blot and figure legend--Revised version/Replication experiment/S3-6 CYP27A1-3.tif]
